# Premature aging syndrome showing random chromosome number instabilities with *CDC20* mutation

**DOI:** 10.1111/acel.13251

**Published:** 2020-10-23

**Authors:** Harumi Fujita, Takashi Sasaki, Tatsuo Miyamoto, Silvia Natsuko Akutsu, Showbu Sato, Takehiko Mori, Kazuhiko Nakabayashi, Kenichiro Hata, Hisato Suzuki, Kenjiro Kosaki, Shinya Matsuura, Yoichi Matsubara, Masayuki Amagai, Akiharu Kubo

**Affiliations:** ^1^ Department of Dermatology Keio University School of Medicine Tokyo Japan; ^2^ KOSÉ Endowed Program for Skin Care and Allergy Prevention Keio University School of Medicine Tokyo Japan; ^3^ Center for Supercentenarian Medical Research Keio University School of Medicine Tokyo Japan; ^4^ Department of Genetics and Cell Biology Research Institute for Radiation Biology and Medicine Hiroshima University Hiroshima Japan; ^5^ Division of Hematology Department of Internal Medicine Keio University School of Medicine Tokyo Japan; ^6^ Department of Maternal‐Fetal Biology National Center for Child Health and Development Tokyo Japan; ^7^ Center for Medical Genetics Keio University School of Medicine Tokyo Japan; ^8^ National Center for Child Health and Development Tokyo Japan

**Keywords:** aging, Cdc20 proteins, chromosomal instability, chromosome segregation, genomic instability, M phase cell cycle checkpoints, premature

## Abstract

Damage to the genome can accelerate aging. The percentage of aneuploid cells, that is, cells with an abnormal number of chromosomes, increases during aging; however, it is not clear whether increased aneuploidy accelerates aging. Here, we report an individual showing premature aging phenotypes of various organs including early hair loss, atrophic skin, and loss of hematopoietic stem cells; instability of chromosome numbers known as mosaic variegated aneuploidy (MVA); and spindle assembly checkpoint (SAC) failure. Exome sequencing identified a *de novo* heterozygous germline missense mutation of c.856C>A (p.R286S) in the mitotic activator *CDC20*. The mutant CDC20 showed lower binding affinity to BUBR1 during the formation of the mitotic checkpoint complex (MCC), but not during the interaction between MCC and the anaphase‐promoting complex/cyclosome (APC/C)–CDC20 complex. While heterozygous knockout of *CDC20* did not induce SAC failure, knock‐in of the mutant *CDC20* induced SAC failure and random aneuploidy in cultured cells, indicating that the particular missense mutation is pathogenic probably via the resultant imbalance between MCC and APC/C‐CDC20 complex. We postulate that accelerated chromosome number instability induces premature aging in humans, which may be associated with early loss of stem cells. These findings could form the basis of a novel disease model of the aging of the body and organs.

## Graphical abstract

We report an individual showing premature aging, instability of chromosome numbers, spindle assembly checkpoint failure, and a heterozygous missense mutation in *CDC20*. The CDC20 mutant showed low binding affinity to BUBR1 in the formation of the mitotic checkpoint complex and induced spindle assembly checkpoint failure in vitro. The case is postulated to be a novel syndrome showing that accelerated chromosome number instability induces premature aging.

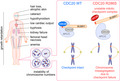

## 1 INTRODUCTION

Premature aging syndromes are rare disorders with clinical features that mimic physiological aging at an early age. The known causative genes are related to the maintenance and repair of genomic DNA (Vijg & Suh, 2013). A failure of their genomic integrity is considered a primary driving force of human aging (Vijg & Suh, 2013).

Aneuploidy, the presence of an abnormal number of chromosomes, is an example of the failure of genomic integrity. The number of aneuploid cells increases with aging in various organs (Duncan et al., 2012; Jacobs et al., 1961; Rehen et al., 2005). However, it is not known whether an increased number of aneuploid cells directly drive aging in humans. To date, three causative genes, *BUBR1*, *CEP57*, and *TRIP13*, have been found to induce mosaic variegated aneuploidy (MVA) syndromes that show random aneuploidy in a significant percentage of somatic cells (Hanks et al., 2004; Snape et al., 2011; Yost et al., 2017). Increased risk for carcinogenesis, congenital abnormalities, including intrauterine growth retardation, and infantile death are major symptoms in those MVA syndromes. Only tissue‐specific premature aging phenotypes, including cataracts, have been reported in those MVA syndromes, while *Bubr1* hypomorphic mice show MVA and systemic premature aging phenotypes, including kyphosis, muscle atrophy, dermal thinning, heart failure, and loss of fat tissues (Baker et al., 2004).

Here, we present a patient exhibiting premature aging of various organs, early stem cell loss, and MVA. We identified a missense variant of *CDC20* that occurred *de novo* and characterized the variant, which induced spindle assembly checkpoint (SAC) failure and MVA. The findings of this case could provide the basis for a novel disease model of human aging.

## 2 RESULTS

**Figure 1:**
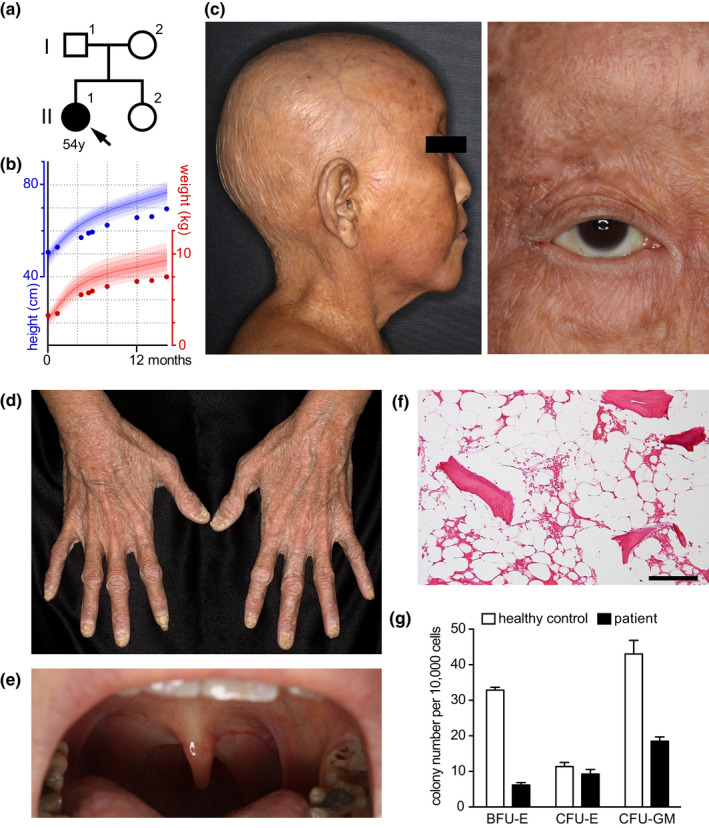
Clinical and cellular phenotypes of the patient. (a) The patient's family tree. (b) The patient's growth curve of height and weight plotted on a standard growth chart of a Japanese girl (Ministry of Health, 2010a, 2010b). Colored lines correspond to the 97th, 90th, 75th, 50th, 25th, 10th, and 3rd percentiles from top to bottom. (c–e) Clinical photographs of the patient showing the loss of scalp hair, eyelashes and eyebrows (c), dry, atrophic skin (d), and atrophic tonsils and uvula (e) at age 51. (f) Hematoxylin and eosin staining of a bone marrow biopsy section. Scale = 100 µm. (g) The numbers of colonies per 1 × 10^4^ bone marrow mononuclear cells in the patient and an age‐matched control (means ± SEM from six technical replicates from each subject). BFU‐E, burst‐forming unit–erythroid; CFU‐E, colony‐forming unit–erythroid; CFU‐GM, colony‐forming unit–granulocytes/macrophages.

The patient, a 54‐year‐old Japanese woman (II‐1 in Figure 1a), was born healthy from healthy parents but gradually developed growth retardation without mental retardation (Figure 1b); dry and atrophic skin with hyper‐ and hypo‐pigmented macules, atrophic uvula, and oral mucosa; and hair loss on the scalp and body before she reached 20 years of age (Figure 1c‐e and Figure S1). From age 20 to 50, she developed bilateral renal atrophy, bilateral cataracts, femur head necrosis, kyphosis, anemia, cardiac insufficiency, and thyroid atrophy (Tables S1 and S2; and Supplemental Text 1). She was cancer‐free until uterine endometrial carcinoma appeared at age 48, which was completely excised and without recurrence 5 years after the operation without adjuvant therapy. Bone marrow biopsy at age 50 revealed replacement of bone marrow by fat tissue and a marked decrease in colony‐forming efficiency of hematopoietic cells (Figure 1f, g). The patient showed no neurodevelopmental, cognitive, memory, or other neurological defects. No immunodeficiency or autoimmunity was identified.

**Figure 2:**
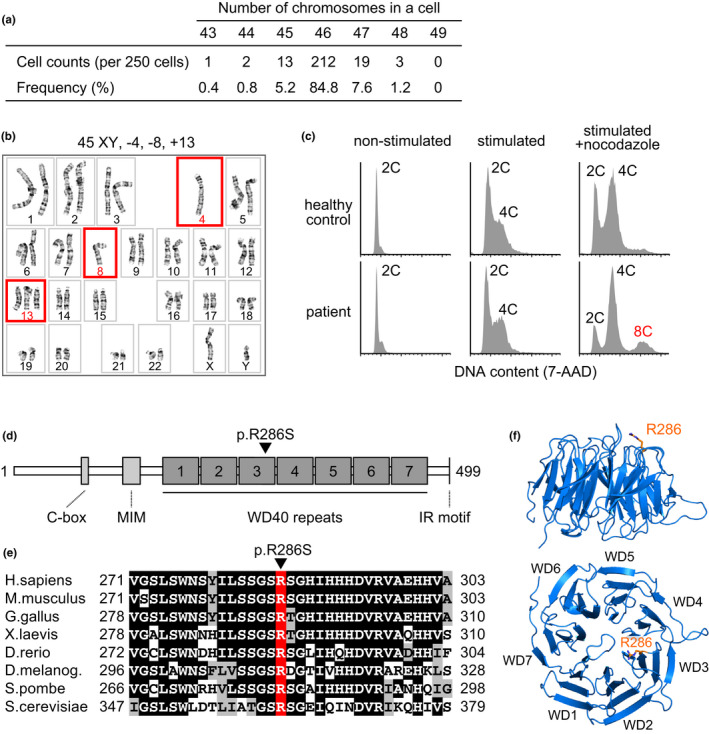
Chromosome number instability and *CDC20* mutation in the patient's peripheral blood mononuclear cells. (a) Chromosome number instability observed in the patient's peripheral blood mononuclear cells (PBMCs). (b) A representative karyotype of the patient's PBMCs. Red squares indicate chromosome gain or loss. (c) Flow‐cytometric analyses of DNA contents in the PBMCs of the patient and a healthy control with or without mitotic stimulation and nocodazole treatment. X‐axes indicate nuclear DNA contents (2C, diploid; 4C, tetraploid; 8C, octoploid) stained with 7‐amino‐actinomycin D (7‐AAD) on a linear scale. (d) A schematic representation of the human CDC20 protein. The R286 amino acid residue is indicated with a black arrowhead. MIM, MAD2 interacting motif. (e) A cross‐species amino acid sequence comparison of CDC20 around the R286 residue (red). Identical and conserved amino acids are marked with black and gray boxes, respectively. (f) Side and top view of the WD40 repeat propeller domains (WD1–7) of the human CDC20 protein. The R286 residue (orange stick) is located at the top of WD3.

The patient showed complete female genitalia but had a history of surgical extrusion of streak gonads and renal failure, and thus, Frasier syndrome was first suspected (Barbaux et al., 1997). Karyotype analyses and genomic sequencing revealed the patient had XY chromosomes but no causative mutations of Frasier syndrome in *WT1* (data not shown). Instead, the patient's PBMCs revealed MVA, a random gain and loss of various chromosomes, in ~15% of cells (38/250 cells, Figure 2a, b; Table S3) with no detectable chromosome breakage (0/250 cells). Premature chromatid separation (PCS) was observed in ~6% of cells (12/200 cells), while this was <2% in a normal cohort (Kajii et al., 1998). Micronuclei, the hallmark of chromosome missegregation, was observed in 2.5% of cells (Table S4, control: 0.7 ± 0.2% [mean ± SEM], *n* = 3). Treatment of the patient's PBMCs with nocodazole, a microtubule‐depolymerizing reagent, showed the accumulation of octoploid cells (Figure 2c), indicating aberrant cell cycle exit from metaphase due to SAC failure. Thus, we diagnosed the patient with MVA syndrome and decided not to administer adjuvant chemotherapy using taxanes after surgery for uterine endometrial carcinoma at the age of 48 years.

Typical symptoms of the known MVA syndromes caused by mutations in *BUBR1*, *CEP57*, and *TRIP13* are intrauterine growth retardation, mental retardation, microcephaly, and childhood cancer (Hanks et al., 2004; Snape et al., 2011; Yost et al., 2017), none of which were observed in the patient (Table S1). Exome sequencing of genomic DNA from the patient revealed no rare variants in the causative genes of known MVA syndromes and no significant accumulation of somatic mutations. Screening for genes related to premature aging syndromes revealed that the patient was a heterozygous carrier of several rare variants for Rothmund–Thomson syndrome and *Fanconi anemia* (Table S5), but these are autosomal‐recessive diseases and their phenotypes differ from those of the patient. Exome sequencing revealed that the patient possessed nine homozygous, hemizygous, or compound heterozygous pairs of rare variants and three *de novo* germline monoallelic variants (Figure S2, Table S6). Among them, we focused on the *de novo* c.856C>A (p.R286S) variant in *CDC20* (RefSeq: NM_001255.3/ NP_001246.2) and homozygous in‐frame deletion of c.862+3G>C (p.N235_N287del) in *CENPT* (RefSeq: NM_025082.4/ NP_079358.3, see Supplemental Text 2). CDC20 is a component of the mitotic checkpoint complex (MCC) and a cofactor of the anaphase‐promoting complex/cyclosome (APC/C) (Yu, 2007). CENPT is a kinetochore‐localizing protein important for kinetochore assembly and function (Nishino et al., 2013). The patient's younger sister was a heterozygous carrier of the *CENPT* variant and did not have the *CDC20* variant (Figure S2). The CENPT variant correctly localized to the kinetochore in the Epstein–Barr virus‐transformed lymphoblastoid cell line of the patient (Figure S3). Because the biallelic defect of *CENPT* causes a syndrome characterized by severe growth failure and microcephaly without chromosome number instabilities (Hung et al., 2017), which differed from the patient's phenotypes (Table S7), we further investigated the *CDC20* variant in this study.

**Figure 3:**
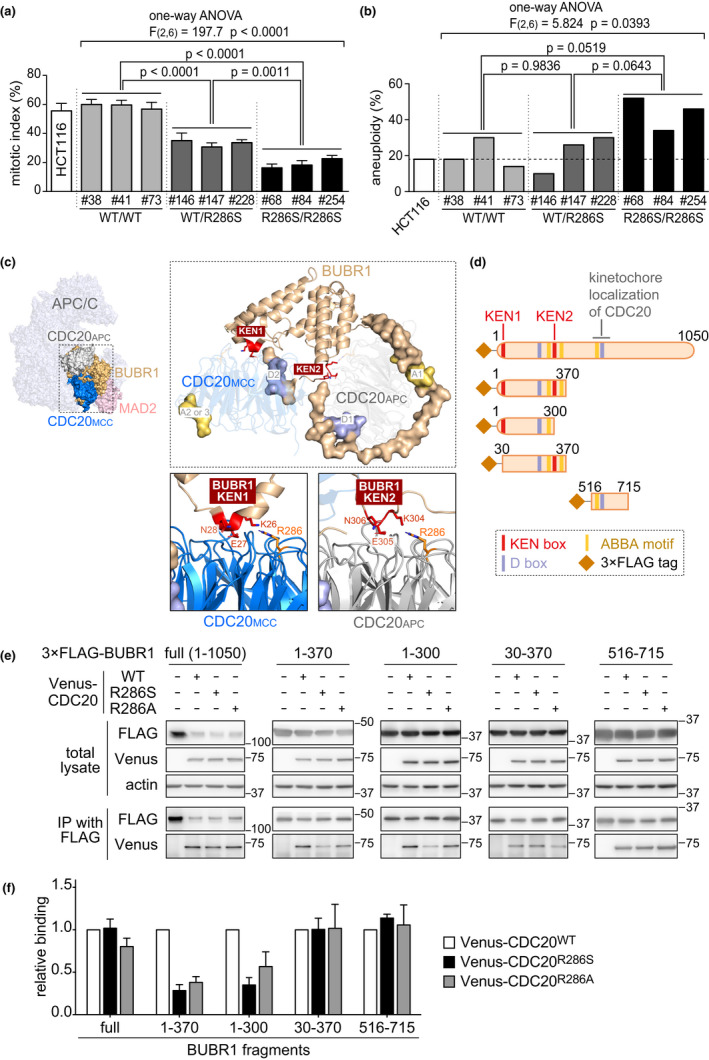
The impact of the CDC20 variant on spindle assembly checkpoint and interaction with BUBR1. (a) The mitotic indices after 12‐h nocodazole treatment of an HCT116 parent clone and its CRISPR‐mediated mutant clones harboring either the wild‐type (WT) silence mutation or the p.R286S mutation in CDC20 (means ± SEM from three independent experiments). The numbers are the code numbers of the mutant clones. (b) The percentage of aneuploid cells in the parent and mutant clones (50 metaphase spreads were counted per clone). (c) The 3D structural model of the mitotic checkpoint complex (MCC) interacting with anaphase‐promoting complex/cyclosome (APC/C) (left panel) through the binding of BUBR1 with CDC20 s consisting of the APC/C‐CDC20 complex and MCC (CDC20_APC_ and CDC20_MCC_, respectively, right upper panel). KEN boxes (KEN1 and KEN2), destruction boxes (D1 and D2), and ABBA motifs (A1, A2, and A3) on BUBR1 are shown with red, blue, and yellow, respectively. The detailed spatial relationships of KEN boxes with the R286 residue of CDC20_MCC_ and CDC20_APC_ are shown in right lower panels. (d) Schematics of the BUBR1 deletion constructs used in immunoprecipitation. Colored lines indicate KEN1, KEN2, destruction boxes (D box; R224STL and R555RPL), and ABBA motifs (I272TVFDE, F340TPYVE, and F528SIFDE). (e) Immunoprecipitation of Venus‐tagged wild type (WT), p.R286S, and p.R286A variants of CDC20 precipitated by 3×FLAG‐tagged BUBR1 deletion constructs shown in d using anti‐FLAG antibody. Upper three rows show Western blotting of the total cell lysates, and lower two rows show Western blotting of the immunoprecipitants. Tags used for the detection and molecular weight indicators (kilodaltons) are shown on the left and right sides of the blot, respectively. The uncropped images are shown in Figure S9. (f) The relative ratios of the band densities for the p.R286S and p.R286A variants of CDC20 compared with WT CDC20 in e (means ± SEM from three independent experiments). *p*‐values, ANOVA/Tukey's test.

CDC20 binds to BUBR1 in the formation of the MCC and in the inhibition of the APC/C via the MCC. The R286 residue of CDC20 locates at the binding site to BUBR1 and is conserved among eukaryotes (Figure 2d‐f); therefore, we examined whether the p.R286S variant affects SAC via knocking in the variation to HCT116 cells (Figure S4). Knocked‐in cells showed allele dosage‐dependent slippage from nocodazole‐induced metaphase arrest and accumulation of octoploid cells (Figure 3a and Figure S5a). By contrast, the induction of a monoallelic frameshift mutation to *CDC20* did not induce SAC failure (Figure S6), suggesting that the p.R286S variant but not haploinsufficiency of *CDC20* specifically induced SAC failure. Furthermore, a significant correlation was observed between the knocked‐in allele dosage of CDC20 p.R286S and aneuploid cell ratios in HCT116 clones (*F*(2,6) = 5.824, *p* = 0.0393 by one‐way ANOVA, Figure 3b and Figure S5b), although aneuploid cells were spontaneously observed in the parent clone of the HCT116 cells. These results indicate the CDC20 p.R286S variant induces SAC failure and MVA.

To determine how the p.R286S variant induces SAC failure, we investigated the interaction between CDC20 and BUBR1. One BUBR1 molecule binds to two CDC20 molecules, namely CDC20_MCC_ and CDC20_APC_; the former is included in the MCC, which consists of BUBR1, CDC20_MCC_, BUB3, and MAD2. The latter is included in the APC/C‐CDC20 complex (APC/C^CDC20^) (Izawa & Pines, 2015). MCC binding to CDC20_APC_ via BUBR1 inhibits the ubiquitination activity of APC/C^CDC20^ and the degradation of APC/C substrates, such as cyclin B1, and therefore blocks cell cycle exit from mitosis (Izawa & Pines, 2015).

**Figure 4:**
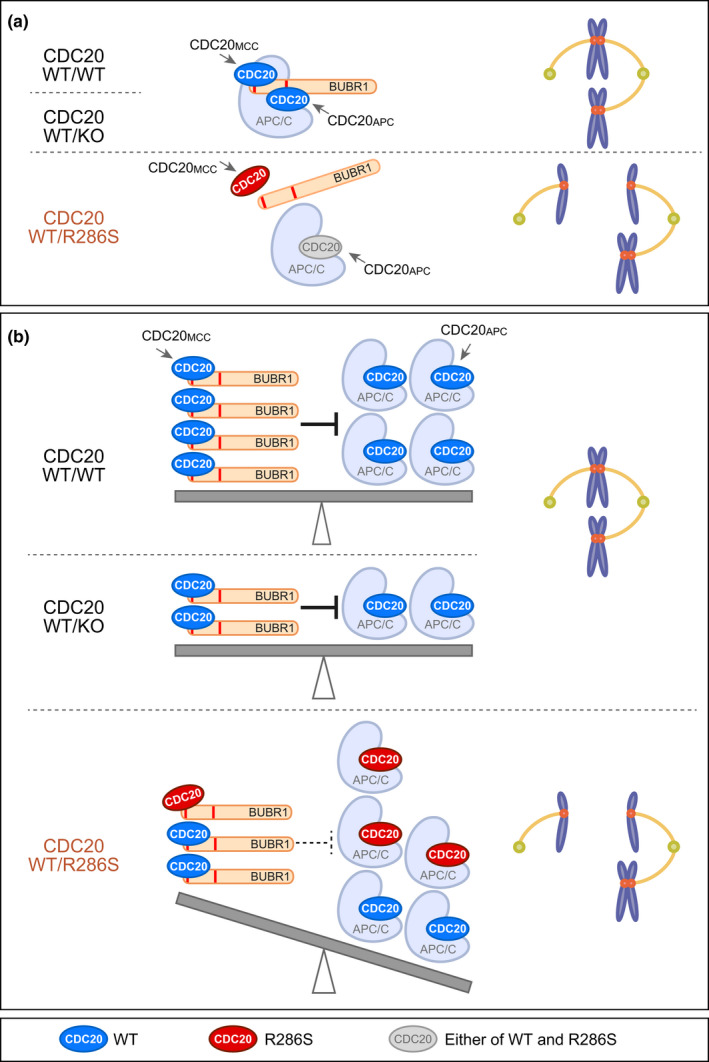
Hypothetical models of the aberrant activation of the APC/C‐CDC20 complex. Hypothetical models of APC/C inhibition by MCC focusing on the interaction of BUBR1 with wild type (blue) and p.R286S variant (red) of CDC20. BUB3 and MAD2 in MCC are not shown for simplification. (a) The increased frequency of stochastic dissociation of the p.R286S variant of CDC20_MCC_ from BUBR1 induces aberrant dissociation of MCC from the APC/C‐CDC20 complex (APC/C^CDC20^), resulting in aberrant entry into anaphase and unequal chromosome segregation (WT/R286S). Haploinsufficiency of CDC20 (WT/KO) does not induce this aberrant dissociation of MCC. (b) Balanced inhibition of the APC/C^CDC20^ by MCC prohibits aberrant entry into anaphase in wild type (WT/WT) and in haploinsufficiency of CDC20 (WT/KO). Reduction in the binding affinity of the CDC20 p.R286S variant to BUBR1 specifically in the formation of MCC but not of the APC/C^CDC20^ induces a molecular imbalance between MCC and the APC/C^CDC20^, resulting in aberrant entry into anaphase (WT/R286S).

The interactions between CDC20 and BUBR1 are mediated by the K26EN and K304EN boxes (KEN1 and KEN2, respectively), destruction (D) boxes, and ABBA motifs of BUBR1 (Figure 3c,d, RefSeq: NM_001211.5/ NP_001202.4) (Alfieri et al., 2016; Di Fiore et al., 2016). Among them, the predicted structural interaction model suggests KEN1 and KEN2 of BUBR1 are faced to the R286 residues of CDC20_MCC_ and CDC20_APC_, respectively (Alfieri et al., 2016) (Figure 3c). An immunoprecipitation assay using FLAG‐tagged BUBR1 fragments and Venus‐tagged CDC20 (Figure 3d‐f) revealed that the N‐terminal BUBR1_1–370_, including both KEN1 and KEN2 showed significant lower binding affinity to the p.R286S variant of CDC20. The binding affinity of the CDC20 variant to full‐length BUBR1 and the BUBR1_516–715_ fragment, a middle part of BUBR1 responsible for CDC20 recruitment to the kinetochores (Lischetti et al., 2014), showed no differences (Figure 3e, f), consistent with the results of the p.R286S variant showing normal kinetochore localization *in vitro* (Figure S7). Remarkably, BUBR1_1–300_ including only KEN1 and BUBR1_1–370_ including both KEN1 and KEN2, but not BUBR1_30–370_ including only KEN2, showed reduced immunoprecipitated CDC20 p.R286S variant when compared with the immunoprecipitation of wild‐type CDC20 (Figure 3e, f). Therefore, the KEN1‐mediated binding to CDC20, but not the KEN2‐mediated binding to another CDC20, was affected in the interaction between BUBR1 and the CDC20 p.R286S variant. The CDC20 p.R286A variant, which had been reported to show lower binding affinity to BUBR1 (Tian et al., 2012) and was used as a control, showed similar specific reduction in the KEN1‐mediated binding between BUBR1 and CDC20 (Figure 3e, f). These results indicate that the R286 residue of CDC20 is specifically responsible for the KEN1‐mediated binding of BUBR1 to CDC20_MCC_, but not KEN2‐mediated binding to CDC20_APC_. The p.R286S CDC20 variant was suggested to induce aberrant activation of the APC/C^CDC20^, possibly via insufficient formation of MCC to inhibit APC/C^CDC20^, resulting in SAC failure and MVA in the patient (Figure 4).

## 3 DISCUSSION

To the best of our knowledge, this is the first reported case of systemic premature aging associated with SAC failure and MVA in humans. The *de novo*‐occurring monoallelic p.R286S missense variant of CDC20 is considered pathogenic. The patient data suggest that aging could be accelerated by increased numbers of aneuploid cells in humans.

Several nonsense mutations of *CDC20* have been registered in human exome databases (dbSNP, 2019). No SAC failure was observed in heterozygous *CDC20* knockout human cells (Figure S6), suggesting that heterozygous *CDC20*‐knockout humans likely exist in the general population without showing SAC failure and MVA phenotype. *Cdc20*‐null mice were embryonic lethal at the two‐cell stage (Li et al., 2007; Manchado et al., 2010). Tamoxifen‐induced conditional knockout of *Cdc20* in mice during the developmental stage revealed severe developmental defects with widespread metaphase arrest in proliferating cells caused by anaphase onset failure (Manchado et al., 2010). In contrast, patient cells investigated in this study showed SAC failure, which accelerate cells to exit metaphase and enter anaphase. Therefore, the cellular phenotypes induced by the monoallelic p.R286S missense mutation of CDC20 are different from the phenotypes induced by CDC20 haploinsufficiency or CDC20 deletion. Our results strongly suggest that a specific missense mutation of the R286 residue of CDC20 induced SAC failure and MVA by affecting the KEN1‐mediated binding of BUBR1 to CDC20_MCC_, but not affecting the KEN2‐mediated binding to CDC20_APC_. This may explain the extreme rarity of the disease because only specific missense mutations on a specific residue may cause the disease.

It is likely that instability of the MCC caused by the lower binding affinity of BUBR1 to the CDC20 p.R286S mutant resulted in insufficient inhibition of the APC/C^CDC20^ by MCC in the patient, for example, via aberrant early dissociation of the MCC from the APC/C^CDC20^ or an imbalance between the MCC and APC/C^CDC20^ ratio (Figure 4). Because the patient has one normal *CDC20* allele, aberrant activation of the APC/C^CDC20^ is supposed to be less frequent in the patient than in the MVA syndrome patients caused by biallelic *BUBR1* mutations. This hypothesis likely explains why the frequency and extent of MVA and PCS were relatively low in the patient compared with *BUBR1*‐deficient MVA syndrome patients (Table S1), and why the patient did not show severe anomalies, childhood cancer, and early death.

The phenotype of systemic premature aging and being XY female were the major clinical differences in our patient compared with known MVA syndromes (Table S1). Exome sequencing revealed no causative genetic defect related to sex determination in the patient. As XO/XY mosaicism can result in the XY female phenotype (Johansen et al., 2012), accidental loss of the Y chromosome due to MVA in the early development of the gonads may have caused the XY female phenotype in our patient. It remains unclear whether systemic premature aging observed in the patient was solely induced by the *CDC20* mutation or by the combination with other genetic factors, for example, the homozygous in‐frame deletion in *CENPT* encoding kinetochore‐localizing protein. The biallelic defect of *CENPT* in humans has been reported to cause severe growth failure and microcephaly but not to cause chromosome number instabilities (Hung et al., 2017), which differed from the phenotypes of our patient. Based on our *in vitro* study, it is likely that SAC failure and MVA in the patient are solely caused by the *CDC20* mutation. If systemic premature aging was also solely induced by the *CDC20* mutation, the disease is considered a novel premature aging syndrome distinct from known MVA syndromes and premature aging syndromes. However, if the patient's phenotype is induced by the combination of *CDC20* mutation and homozygous in‐frame deletion mutations of *CENPT*, the phenotype is considered unique to the patient. Other patients are required to determine whether the systemic premature aging phenotype is solely induced by the *CDC20* mutation.

Several SAC failure mouse models, including *Bubr1* hypomorphic mice, develop systemic premature aging without severe congenital anomalies and pediatric cancer (Baker et al., 2004, 2006; Wijshake et al., 2012). Various cellular stresses, including proteotoxic and metabolic stresses, are known to be induced by aneuploidy in *in vitro* cultured cells (Santaguida & Amon, 2015). Early exhaustion of stem cells has been observed in tissue‐specific knockout mice of Mad2, a component to form MCC with Bubr1 and Cdc20_MCC_ (Foijer et al., 2013; Kollu et al., 2015). *Bubr1* hypomorphic mice showed exhaustion of hematopoietic stem cells after serial transplantation of bone marrow cells (Pfau et al., 2016). The various premature aging phenotypes observed in the patient are likely associated with exhaustion of stem cells in highly proliferating tissues, for example, early hair loss and bone marrow hypoplasia with decreased colony‐forming efficiency of hematopoietic cells, and are associated with cellular stresses in slowly proliferating tissues, for example, kidney and thyroid failure, and cataracts.

The reason why only partial premature aging phenotypes but not systemic premature aging have been observed in the known human MVA syndromes including *BUBR1* deficiency remains unclear. One possible explanation is that the premature aging phenotypes in MVA syndromes are difficult to observe in humans because they have a long lifespan but develop lethal phenotypes including childhood cancer much earlier than the development of premature aging phenotypes. In this context, it is possible that the systemic premature aging phenotypes observed in our patient were induced by MVA and could be observed because the patient did not develop cancer until age 48. Another possible explanation is that the systemic premature aging phenotype is related to the non‐mitotic function of the APC/C^CDC20^ associated with the maintenance of stem cells, which is shown in a model of cancer stem cells where APC/C^CDC20^ is associated with self‐renewal activity of stem cells by interacting with pluripotency‐related transcription factor SOX2 (Mao et al., 2015). Further studies are required to evaluate the non‐mitotic function of the APC/C^CDC20^ in the context of stem cell maintenance.

The present case suggests that SAC failure and chromosome number instabilities can accelerate aging in humans and is suggestive of a vicious cycle between increased aneuploid cells and aging. Further studies on the CDC20 p.R286S variant and other MVA syndrome model animals could form the basis of a novel disease model to investigate aging of the body and organs.

## 4 EXPERIMENTAL PROCEDURES

### 4.1 Ethics statement

This study was approved by the Medicine Ethics Committee of the Keio University School of Medicine. Written informed consent was obtained from the patient, the parents, and the younger sister according to the guidelines of the Institutional Review Board of Keio University School of Medicine. The patient gave written informed consent to publish the clinical images.

### 4.2 Extraction of genomic DNA

Genomic DNA in blood samples was isolated using the Wizard® Genomic DNA Purification Kit (Promega). Genomic DNA from patient saliva was isolated using the Oragene DNA Collection Kit (Kyodo International).

### 4.3 Exome sequencing

Exome sequencing was performed as described previously (Kubo et al., 2013; Takenouchi et al., 2015, 2016). Exome sequencing produced ~90,000,000 paired reads per sample, of which ~99.76% were mapped to the hs37d5 exon region of the human genome sequence assembly. The average coverage of the targeted exonic region was 97.22×, with more than 98.94% of targeted bases covered at over 10× reads. Candidate variants were screened using the autosomal dominant model (*de novo* mutations identified in the patient, but not in the parents) with an MAF <0.001 and the autosomal‐recessive model (homozygous, hemizygous, or compound heterozygous rare variants identified in the patient) with an MAF <0.03 in public databases including the Human Genetic Variation Database (Higasa et al., 2016), the Tohoku Medical Megabank Organization 3.5KJPNv2 (Tadaka et al., 2018), Japanese Genotype‐phenotype Archive (Kodama et al., 2015), 1000 Genomes Project (Genomes Project et al., 2015), and Exome Aggregation Consortium (Lek et al., 2016).

### 4.4 Sanger sequencing

The target DNA fragments were amplified by PCR using HotStarTaq DNA Polymerase (Qiagen) and subjected to direct Sanger sequencing. The sequences of PCR primer pairs are listed in Table S8.

### 4.5 Culture of cell lines

The 293 T cell line, HCT116 cell line, and its derived clones were cultured in Dulbecco's modified Eagle's medium (DMEM; Thermo Fisher Scientific) supplemented with 10% fetal bovine serum (FBS; Bovogen, Keilor East, Australia), 100 units ml^−1^ penicillin, and 100 μg mL^−1^ streptomycin (Wako Pure Chemical). Epstein–Barr virus‐transformed lymphoblastoid cell lines (LCLs) were established from the peripheral blood of the patient and the healthy control by SRL Inc. and cultured in RPMI 1640 medium (Thermo Fisher Scientific) supplemented with 10% FBS, 100 units ml^−1^ penicillin, 100 μg/ml streptomycin, and 0.25 μg/ml amphotericin B (Sigma). Each cell line was maintained at 95% humidified air, 5% CO_2_, and 37°C.

### 4.6 Plasmid construction

For knock‐in of the *CDC20* p.R286S missense mutation into HCT116 cells by CRISPR/Cas9, oligonucleotides encoding single‐guide RNAs (sgRNAs) against *CDC20* were inserted into the pSpCas9(BB)‐2A‐Puro (px459) vector (Addgene). The sequences of sgRNAs were as follows: sgRNA‐E2 target sequence: 5′‐TCCTCCAGTGGTTCACGTTC‐3′; sgRNA‐E3 target sequence: 5′‐GAACATCATGGTGGTGGATG‐3′. pSpCas9(BB)‐2A‐Puro (PX459) V2.0 was a gift from Feng Zhang (Addgene plasmid # 62988; http://n2t.net/addgene:62988; RRID:Addgene_62988) (Ran et al., 2013).

To construct the pCAGGS‐Venus‐hCDC20 plasmid encoding, the fusion protein of Venus (Nagai et al., 2002) and human CDC20, the human *CDC20* open reading frame (ORF) was isolated from the pCMV7.1–3×FLAG‐CDC20 plasmid (Miyamoto et al., 2011) by EcoRI digestion and subcloned into the pCAGGS vector (Niwa et al., 1991) with N‐terminal Venus. The p.R286S mutation was inserted into this plasmid using the CDC20‐QC‐T/B primer pair and the QuikChange Site‐Directed Mutagenesis Kit (Stratagene). The p.R286A mutation was inserted using the CDC20 (KOD‐R286A)‐F/R primer pair and the KOD‐Plus Mutagenesis Kit (Toyobo).

To construct the pCMV7.1–3×FLAG‐hBUBR1 plasmid encoding 3×FLAG‐tagged human BUBR1, human *BUBR1* ORF was PCR‐amplified from the pEGFP‐hBUBR1 plasmid (Miyamoto et al., 2011) using a primer set with a Kpn1 site at the 5′‐end and a BamHI site at the 3’‐end and then was subcloned into the pCMV7.1–3×FLAG vector (Sigma). To construct the plasmids encoding the 3×FLAG‐tagged truncated human BUBR1 proteins, *BUBR1* fragments were PCR‐amplified from pCMV7.1–3×FLAG‐hBUBR1 using primer sets with a BamHI site at the 5′‐end and a NotI site at the 3′‐end. These fragments were subcloned into a modified pCMV7.1–3×FLAG vector, which had a HindIII‐BamHI‐NotI‐SmaI multi‐cloning site.

All constructs used in this study were verified by Sanger sequencing. The primer sequences are listed in Table S8. The molecular weight and antibody reactivity of the product proteins were verified by Western blotting (Figure S8).

### 4.7 CRISPR/Cas9‐mediated knock‐in of the mutation

A single‐cell suspension of 1 × 10^6^ HCT116 cells was electroporated with 2.5 µg px459 vector containing the sgRNA against *CDC20* and 2 µl 100 μM ssODN by Nucleofector II (Lonza). To prevent further digestion of the knock‐in allele by Cas9, four silent mutations were inserted into each ssODNs. These ssODNs included BamHI and BspEI sites for genotyping (Figure S4). The sequences used were as follows: *CDC20 *WT ssODN for sgRNA‐E2: 5’‐GCTCTGGCTTGCTTGCATTTGGTGCTGCCACAGAACCTGATTCCCTTCTTTCCTCCTCCAGTGGATCCCGTTCCGGACACATCCACCACCATGATGTTCGGGTAGCAGAACACCATGTGGCCACACTGAGTGGCCACAGCCAGGAAGTGT‐3’; *CDC20* p.R286S mutant ssODN for sgRNA‐E2: 5’‐GCTCTGGCTTGCTTGCATTTGGTGCTGCCACAGAACCTGATTCCCTTCTTTCCTCCTCCAGTGGATCCAGTTCCGGACACATCCACCACCATGATGTTCGGGTAGCAGAACACCATGTGGCCACACTGAGTGGCCACAGCCAGGAAGTGT‐3’; *CDC20* WT ssODN for sgRNA‐E3: 5’‐CTGGCTTGCTTGCATTTGGTGCTGCCACAGAACCTGATTCCCTTCTTTCCTCCTCCAGTGGATCCCGTTCTGGTCATATCCACCACCATGATGTTCGGGTAGCAGAACACCATGTGGCCACACTGAGTGGCCACAGCCAGGAAGTGTGTG‐3’; *CDC20* p.R286S mutant ssODN for sgRNA‐E3: 5’‐CTGGCTTGCTTGCATTTGGTGCTGCCACAGAACCTGATTCCCTTCTTTCCTCCTCCAGTGGATCCAGTTCTGGTCATATCCACCACCATGATGTTCGGGTAGCAGAACACCATGTGGCCACACTGAGTGGCCACAGCCAGGAAGTGTGTG‐3.’

The transfected cells were plated onto two 100‐mm dishes. After 24‐h incubation, the cells were treated with puromycin (0.75 μg/ml) for 48 h and then further incubated with puromycin‐free media for 2 weeks, after which the colonies were picked. To screen for positive clones, genomic DNA was extracted from each clone by the phenol/chloroform method, and the target locus was amplified by PCR using HotStarTaq and the *CDC20* ex8F/R primer pair. PCR products were further digested with BamHI or BspEI restriction enzymes and electrophoresed on agarose gels. Positive clones were further confirmed by direct Sanger sequencing.

### 4.8 Chromosome analyses

Karyotype analyses and premature chromatid separation analyses of the patient blood sample were performed at the LSI Medience Corporation. For chromosome count analyses of the HCT116 clones, the cell cycle was arrested with 0.12 μg/ml colcemid for 1 h and then treated with 8 ml 0.075 M KCl at room temperature for 20 min. We added 2 ml Carnoy's solution, and the cells were incubated for 10 min and washed two or three times with Carnoy's solution. The chromosomes were spread onto glass slides using a HANABI metaphase spreader (ADSTEC).

### 4.9 Cytokinesis‐block micronucleus (CBMN) assay

The CBMN assay was performed as described previously (Fenech, 2007). Briefly, PBMCs were isolated from the peripheral blood of the patient and healthy controls by gradient centrifugation using Lymphoprep (Axis‐Shield) and cultured in RPMI 1640 supplemented with 10% FBS, 100 units ml penicillin, 100 μg/ml streptomycin, and 30 μg/ml phytohemagglutinin (Wako Pure Chemical) at 95% humidified air, 5% CO_2_, and 37°C. After 44 h, cytochalasin B (Sigma) was added, and the cells were further cultured for 22–26 h. The cells were smeared onto glass slides, stained with May–Grünwald–Giemsa, and mounted with DPX mounting medium (Sigma). More than 1000 binucleated cells were examined under a BIOREVO microscope BZ‐9000 (Keyence) at ×1000 magnification to calculate the frequency of micronucleus formation.

### 4.10 Colony‐forming unit assay of bone marrow cells

The patient bone marrow cells were obtained from the remainder of the bone marrow aspiration sample, and mononuclear cells were isolated by gradient centrifugation using Lymphoprep (Axis‐Shield). An age‐matched normal control bone marrow mononuclear cell sample was obtained from AllCells. Cells were cultured in MethoCult H4034 Optimum (STEMCELL Technologies) at 1 × 10^4^ or 2 × 10^4^ cells per 35 mm dish at 95% humidified air, 5% CO_2_, and 37°C for 14 days, after which the colony numbers were counted.

### 4.11 Mitotic checkpoint analyses

For the mitotic index analyses of the HCT116 cell clones, cells were grown on coverslips (Matsunami Glass), coated with collagen (Cellmatrix; Nitta Gelatin), and treated with 200 ng/ml nocodazole (Sigma). Following incubation for 0 or 12 h, the cells were fixed with 4% paraformaldehyde (Muto Pure Chemicals, Tokyo, Japan) for 15 min at room temperature, and the DNA was stained with Hoechst 33258 or 33342 (Thermo Fisher Scientific), before being mounted with Mowiol (Merck). At least 200 cells were analyzed under a confocal laser scanning microscope LSM710 (Carl Zeiss) at ×400 magnification.

To analyze the DNA content of the PBMCs, mononuclear cells were obtained from the peripheral blood by gradient centrifugation using Lymphoprep (Axis‐Shield) and suspended in RPMI 1640 medium supplemented with 10% FBS, 100 units ml penicillin, and 100 μg/ml streptomycin. The cells were stimulated with 50 ng/ml phorbol 12‐myristate 13‐acetate (Sigma) and 1 μg/ml ionomycin (Wako Pure Chemical) at 95% humidified air, 5% CO_2_, and 37°C for 4 days in 96‐well round‐bottom plates. Next, 25 ng/ml nocodazole was added, and the cells were cultured for another 4 days. Harvested cells were stained with Live/Dead Fixable Aqua Dead Cell Stains (Thermo Fisher Scientific) and fixed with 4% paraformaldehyde (Muto Pure Chemicals) for 15 min on ice. Then the cells were permeabilized with 0.1% Triton X‐100 in PBS for 5 min, washed with PBS, and stained with 15 μg/ml 7‐AAD for 3 h at room temperature. The DNA contents were analyzed using a FACSCanto (Becton Dickinson) flow cytometer. For DNA content analyses of the HCT116 cell clones, the cells were treated with 200 ng/ml nocodazole (Sigma) for 36 h. The harvested cells were processed and analyzed in the same way as the PBMCs.

### 4.12 Immunoprecipitation analyses

The 293 T cells were plated onto a 35‐mm dish and transiently transfected with plasmids encoding 3×FLAG‐tagged truncated BUBR1 fragments and Venus‐CDC20 fusion proteins by Lipofectamine LTX with PLUS reagent (Thermo Fisher Scientific). One day after transfection, the cells were lysed with Triton X‐100‐based lysis buffer (0.5% Triton X‐100, 150 mM NaCl, 20 mM Tris‐HCl (pH 7.5), 1 mM EDTA with a 1/100 concentration of protease inhibitor cocktail (Sigma)). The lysates were sheared with a 23 G needle, incubated on ice for 30 min, and centrifuged at 20,000 × *g* for 10 min at 4°C. The supernatants were incubated with anti‐FLAG M2 Affinity Gel (P8340, Sigma) for 90 min at 4°C with constant rotation. The beads were washed three times with lysis buffer, boiled in SDS‐sample buffer for 5 min, and subjected to SDS‐PAGE and immunoblotting. All images were acquired with an LAS‐4000 mini (Fujifilm, Tokyo, Japan/GE Healthcare) and displayed without altering the brightness or contrast. Band densities were calculated with ImageQuant TL software (version 8.1, GE Healthcare). The following antibodies were used at the indicated dilutions: mouse anti‐DDDDK‐tag (M‐185–3, Medical and Biological Laboratories [MBL]) 1:10,000; mouse anti‐GFP (04363–66, Nacalai Tesque, Kyoto, Japan) 1:2000; rabbit anti‐CDC20 p55 CDC (H‐175, Santa Cruz Biotechnology, Dallas, TX, USA) 1:200; mouse anti‐BUBR1 (K0169–3, MBL) 1:1000; mouse anti‐beta‐actin (M177–3, MBL) 1:1000; goat anti‐rabbit IgG‐peroxidase conjugate (A0545, Sigma) 1:50,000; and goat anti‐mouse IgG‐peroxidase conjugate (P0447, Dako, Glostrup, Denmark) 1:1500. The uncropped images are shown in Figure S9.

### 4.13 Fluorescence imaging analyses of cultured cells

To analyze the localization of the Venus‐CDC20 fusion protein in the HCT116 cell line, cells were seeded onto coverslips (Matsunami Glass) coated with poly‐L‐lysine (Sigma) and transiently transfected with plasmids encoding WT or p.R286S mutant CDC20 protein fused to Venus fluorescent protein (Nagai et al., 2002) by Lipofectamine LTX with PLUS reagent (Thermo Fisher Scientific). At 24–48 h after transfection, the cells were fixed with 4% paraformaldehyde (Muto Pure Chemicals) for 15 min at room temperature. The DNA was stained with Hoechst 33258 (Thermo Fisher Scientific) for 30 min at room temperature, and the samples were mounted with Mowiol (Merck).

For the immunofluorescent staining of the CENPT protein, patient and healthy control LCLs were fixed with 2% paraformaldehyde (Muto Pure Chemicals) for 15 min at 37°C and smeared on glass slides. For membrane permeabilization, the slides were soaked in 0.1% Triton X‐100 in PBS for 15 min at room temperature, and nonspecific binding was blocked with 10% newborn calf serum (Thermo Fisher Scientific) and 5% normal goat serum (Dako) in PBS for >1 h at room temperature. Then, the slides were incubated with the primary antibodies for 1 h at room temperature, washed in PBS, and reacted with fluorescence‐conjugated secondary antibodies and Hoechst 33342 for 1 h at room temperature. Then, the slides were washed in PBS and mounted with Mowiol (Merck).

The images were acquired on a confocal laser scanning microscope LSM710 (Carl Zeiss) using a Plan‐Apochromat 63×/1.40 oil DIC M27 objective with a working distance of 0.19 mm, a 405 nm diode laser (30 mW), a 488 nm Argon laser (25/35 mW), and a 543 nm He‐Ne laser (1 mW). All images were processed with Imaris software (version 8.2.0; Bitplane, Zurich, Switzerland), Photoshop CS4 (Adobe), and Illustrator CS6 (Adobe). The following antibodies and reagents were used at the indicated dilutions: rat anti‐CENP‐T (D286–3, MBL) 1:200; rabbit anti‐alpha tubulin (ab52866, Abcam) 1:500; goat anti‐rat IgG‐Alexa Fluor 568 conjugate (ab175710, Abcam) 1:200; goat anti‐rabbit IgG‐Alexa Fluor 488 conjugate (A‐11034, Thermo Fisher Scientific) 1:200; and Hoechst 33258 or Hoechst 33342 (Thermo Fisher Scientific) 1:1000.

### 4.14 RT‐PCR

Total RNA was extracted from the patient and healthy control LCLs with TRIzol reagent (Thermo Fisher Scientific) and an RNeasy Mini Kit (Qiagen), and then subjected to DNase treatment (RNase‐Free DNase Set; Qiagen). Complementary DNA synthesis was performed with ReverTra Ace (Toyobo), and a subsequent PCR was performed with HotStarTaq DNA Polymerase (Qiagen). The primer sequences are described in Table S8.

### 4.15 Statistics

Statistical significance of the differences in the mitotic indices and the percentages of aneuploid cells between CRISPR‐mediated HCT116 mutant clones shown in Figure 3 were analyzed with a one‐way analysis of variance (ANOVA) and the *post hoc* Tukey's multiple comparison test using Prism software (ver.6; GraphPad Software).

### 4.16 Protein sequence alignment

Cross‐species CDC20 protein sequence alignment was generated using the ClustalW program (https://www.genome.jp/tools‐bin/clustalw) (Li, 2003) and the BoxShade 3.21 program (https://embnet.vital‐it.ch/software/BOX_form.html). We used the following protein sequences for comparison: *Homo sapiens* CDC20 (NP_001246.2), *Mus musculus* CDC20 (NP_075712.2), *Gallus gallus* CDC20 (NP_001006536.1), *Xenopus laevis* Cdc20.s (NP_001079443.1), *Danio rerio* Cdc20 (NP_998245.2), *Drosophila melanogaster* Fizzy/Cdc20 (NP_477501.1), *Schizosaccaromyces pombe* Slp1/Cdc20 (NP_593161.1), and *Saccharomyces cerevisiae* Cdc20 (NP_011399.1).

### 4.17 3D modeling of the protein structure

All 3D images of the protein structures and their interactions were generated using the PyMOL Molecular Graphics System, Version 2.0.7 (Schrödinger LLC) (https://pymol.org). For Figure 2f, the 3D structural model of CDC20 was reconstituted from the published Protein Data Bank (PDB) file ID: 4GGD (Tian et al., 2012). For Figure 3c, the 3D structural model of the APC/C‐CDC20‐MCC interaction was reconstituted from the published PDB file ID: 5LCW (Alfieri et al., 2016).

## CONFLICT OF INTEREST

The authors declare that they have no conflict of interest.

## AUTHOR CONTRIBUTIONS

AK conceptualized the study. TS and KN Curated the data. TS, KN, KH, and HS involved in formal analysis. HF, KK, YM, MA, and AK acquired funding. HF, TMi, SNA, TMo, and AK investigated the study. HF, TMi, and SNA designed the methodology. MA and AK administered the project. HF, TMi, SS, TMo, and AK provided resources. MA and AK supervised the study. HF and AK validated and visualized the data, and wrote the original draft. TMi, SM, MA, and AK reviewed and edited the manuscript.

## Data Availability

The data that support the findings of this study are available from the corresponding author upon reasonable request.

## References

[acel13251-bib-0001] Alfieri, C. , Chang, L. , Zhang, Z. , Yang, J. , Maslen, S. , Skehel, M. , & Barford, D. (2016). Molecular basis of APC/C regulation by the spindle assembly checkpoint. Nature, 536(7617), 431–436. 10.1038/nature19083 27509861 PMC5019344

[acel13251-bib-0002] Baker, D. J. , Jeganathan, K. B. , Cameron, J. D. , Thompson, M. , Juneja, S. , Kopecka, A. , Kumar, R. , Jenkins, R. B. , de Groen, P. C. , Roche, P. , & van Deursen, J. M. (2004). BubR1 insufficiency causes early onset of aging‐associated phenotypes and infertility in mice. Nature Genetics, 36(7), 744–749. 10.1038/ng1382 15208629

[acel13251-bib-0003] Baker, D. J. , Jeganathan, K. B. , Malureanu, L. , Perez‐Terzic, C. , Terzic, A. , & van Deursen, J. M. (2006). Early aging‐associated phenotypes in Bub3/Rae1 haploinsufficient mice. Journal of Cell Biology, 172(4), 529–540. 10.1083/jcb.200507081 16476774 PMC2063673

[acel13251-bib-0004] Barbaux, S. , Niaudet, P. , Gubler, M.‐C. , Grünfeld, J.‐P. , Jaubert, F. , Kuttenn, F. , Fékété, C. N. , Souleyreau‐Therville, N. , Thibaud, E. , Fellous, M. , & McElreavey, K. (1997). Donor splice‐site mutations in WT1 are responsible for Frasier syndrome. Nature Genetics, 17(4), 467–470. 10.1038/ng1297-467 9398852

[acel13251-bib-0005] Database of Single Nucleotide Polymorphisms (dbSNP) (2019). Bethesda (MD): National Center for Biotechnology Information, National Library of Medicine. dbSNP gene ID:991, (dbSNP Build ID: 153 (Aug 8, 2019)). Retrieved from https://www.ncbi.nlm.nih.gov/SNP/snp_ref.cgi?locusId=991

[acel13251-bib-0006] Di Fiore, B. , Wurzenberger, C. , Davey, N. E. , & Pines, J. (2016). The mitotic checkpoint complex requires an evolutionary conserved cassette to bind and inhibit active APC/C. Molecular Cell, 64(6), 1144–1153. 10.1016/j.molcel.2016.11.006 27939943 PMC5179498

[acel13251-bib-0007] Duncan, A. W. , Hanlon Newell, A. E. , Smith, L. , Wilson, E. M. , Olson, S. B. , Thayer, M. J. , Strom, S. C. , & Grompe, M. (2012). Frequent aneuploidy among normal human hepatocytes. Gastroenterology, 142(1), 25–28. 10.1053/j.gastro.2011.10.029 22057114 PMC3244538

[acel13251-bib-0008] Fenech, M. (2007). Cytokinesis‐block micronucleus cytome assay. Nature Protocols, 2(5), 1084–1104. 10.1038/nprot.2007.77 17546000

[acel13251-bib-0009] Foijer, F. , DiTommaso, T. , Donati, G. , Hautaviita, K. , Xie, S. Z. , Heath, E. , Smyth, I. , Watt, F. M. , Sorger, P. K. , & Bradley, A. (2013). Spindle checkpoint deficiency is tolerated by murine epidermal cells but not hair follicle stem cells. Proceedings of the National Academy of Sciences United States of America, 110(8), 2928–2933. 10.1073/pnas.1217388110 PMC3581953 23382243

[acel13251-bib-0010] Genomes Project C. , Auton, A. , Brooks, L. D. , Durbin, R. M. , Garrison, E. P. , Kang, H. M. , & Abecasis, G. R. (2015). A global reference for human genetic variation. Nature, 526(7571), 68–74. 10.1038/nature15393 26432245 PMC4750478

[acel13251-bib-0011] Hanks, S. , Coleman, K. , Reid, S. , Plaja, A. , Firth, H. , FitzPatrick, D. , Kidd, A. , Méhes, K. , Nash, R. , Robin, N. , Shannon, N. , Tolmie, J. , Swansbury, J. , Irrthum, A. , Douglas, J. , & Rahman, N. (2004). Constitutional aneuploidy and cancer predisposition caused by biallelic mutations in BUB1B. Nature Genetics, 36(11), 1159–1161. 10.1038/ng1449 15475955

[acel13251-bib-0012] Higasa, K. , Miyake, N. , Yoshimura, J. , Okamura, K. , Niihori, T. , Saitsu, H. , Doi, K. , Shimizu, M. , Nakabayashi, K. , Aoki, Y. , Tsurusaki, Y. , Morishita, S. , Kawaguchi, T. , Migita, O. , Nakayama, K. , Nakashima, M. , Mitsui, J. , Narahara, M. , Hayashi, K. , … Matsuda, F. (2016). Human genetic variation database, a reference database of genetic variations in the Japanese population. Journal of Human Genetics, 61(6), 547–553. 10.1038/jhg.2016.12 26911352 PMC4931044

[acel13251-bib-0013] Hung, C. Y. , Volkmar, B. , Baker, J. D. , Bauer, J. W. , Gussoni, E. , Hainzl, S. , Klausegger, A. , Lorenzo, J. , Mihalek, I. , Rittinger, O. , Tekin, M. , Dallman, J. E. , & Bodamer, O. A. (2017). A defect in the inner kinetochore protein CENPT causes a new syndrome of severe growth failure. PLoS One, 12(12), e0189324 10.1371/journal.pone.0189324 29228025 PMC5724856

[acel13251-bib-0014] Izawa, D. , & Pines, J. (2015). The mitotic checkpoint complex binds a second CDC20 to inhibit active APC/C. Nature, 517(7536), 631–634. 10.1038/nature13911 25383541 PMC4312099

[acel13251-bib-0015] Jacobs, P. A. , Court Brown, W. M. , & Doll, R. (1961). Distribution of human chromosome counts in relation to age. Nature, 191, 1178–1180. 13718573 10.1038/1911178a0

[acel13251-bib-0016] Kajii, T. , Kawai, T. , Takumi, T. , Misu, H. , Mabuchi, O. , Takahashi, Y. , Tachino, M. , Nihei, F. , & Ikeuchi, T. (1998). Mosaic variegated aneuploidy with multiple congenital abnormalities: homozygosity for total premature chromatid separation trait. American Journal of Medical Genetics, 78(3), 245–249. 9677059 10.1002/(sici)1096-8628(19980707)78:3<245::aid-ajmg7>3.0.co;2-o

[acel13251-bib-0017] Kodama, Y. , Mashima, J. , Kosuge, T. , Katayama, T. , Fujisawa, T. , Kaminuma, E. , Ogasawara, O. , Okubo, K. , Takagi, T. , & Nakamura, Y. (2015). The DDBJ Japanese Genotype‐phenotype Archive for genetic and phenotypic human data. Nucleic Acids Research, 43(Database issue), D18–22. 10.1093/nar/gku1120 25477381 PMC4383935

[acel13251-bib-0018] Kollu, S. , Abou‐Khalil, R. , Shen, C. , & Brack, A. S. (2015). The spindle assembly checkpoint safeguards genomic integrity of skeletal muscle satellite cells. Stem Cell Reports, 4(6), 1061–1074. 10.1016/j.stemcr.2015.04.006 25960061 PMC4471836

[acel13251-bib-0019] Kubo, A. , Shiohama, A. , Sasaki, T. , Nakabayashi, K. , Kawasaki, H. , Atsugi, T. , Sato, S. , Shimizu, A. , Mikami, S. , Tanizaki, H. , Uchiyama, M. , Maeda, T. , Ito, T. , Sakabe, J.‐I. , Heike, T. , Okuyama, T. , Kosaki, R. , Kosaki, K. , Kudoh, J. , … Amagai, M. (2013). Mutations in SERPINB7, encoding a member of the serine protease inhibitor superfamily, cause Nagashima‐type palmoplantar keratosis. American Journal of Human Genetics, 93(5), 945–956. 10.1016/j.ajhg.2013.09.015 24207119 PMC3824127

[acel13251-bib-0020] Lek, M. , Karczewski, K. J. , Minikel, E. V. , Samocha, K. E. , Banks, E. , Fennell, T. , O’Donnell‐Luria, A. H. , Ware, J. S. , Hill, A. J. , Cummings, B. B. , Tukiainen, T. , Birnbaum, D. P. , Kosmicki, J. A. , Duncan, L. E. , Estrada, K. , Zhao, F. , Zou, J. , Pierce‐Hoffman, E. , Berghout, J. , … MacArthur, D. G. (2016). Analysis of protein‐coding genetic variation in 60,706 humans. Nature, 536(7616), 285–291. 10.1038/nature19057 27535533 PMC5018207

[acel13251-bib-0021] Li, K. B. (2003). ClustalW‐MPI: ClustalW analysis using distributed and parallel computing. Bioinformatics, 19(12), 1585–1586. 10.1093/bioinformatics/btg192 12912844

[acel13251-bib-0022] Li, M. , York, J. P. , & Zhang, P. (2007). Loss of Cdc20 causes a securin‐dependent metaphase arrest in two‐cell mouse embryos. Molecular and Cellular Biology, 27(9), 3481–3488. 10.1128/MCB.02088-06 17325031 PMC1899968

[acel13251-bib-0023] Lindhardt Johansen, M. , Hagen, C. P. , Rajpert‐De Meyts, E. , Kjærgaard, S. , Petersen, B. L. , Skakkebæk, N. E. , Main, K. M. , & Juul, A. (2012). 45, X/46, XY mosaicism: Phenotypic characteristics, growth, and reproductive function–a retrospective longitudinal study. Journal of Clinical Endocrinology and Metabolism, 97(8), E1540–1549. 10.1210/jc.2012-1388 22605431

[acel13251-bib-0024] Lischetti, T. , Zhang, G. , Sedgwick, G. G. , Bolanos‐Garcia, V. M. , & Nilsson, J. (2014). The internal Cdc20 binding site in BubR1 facilitates both spindle assembly checkpoint signalling and silencing. Nature Communications, 5, 5563 10.1038/ncomms6563 25482201

[acel13251-bib-0025] Manchado, E. , Guillamot, M. , de Carcer, G. , Eguren, M. , Trickey, M. , Garcia‐Higuera, I. , & Malumbres, M. (2010). Targeting mitotic exit leads to tumor regression in vivo: Modulation by Cdk1, Mastl, and the PP2A/B55alpha, delta phosphatase. Cancer Cell, 18(6), 641–654. 10.1016/j.ccr.2010.10.028 21156286

[acel13251-bib-0026] Mao, D. D. , Gujar, A. D. , Mahlokozera, T. , Chen, I. , Pan, Y. , Luo, J. , Brost, T. , Thompson, E. A. , Turski, A. , Leuthardt, E. C. , Dunn, G. P. , Chicoine, M. R. , Rich, K. M. , Dowling, J. L. , Zipfel, G. J. , Dacey, R. G. , Achilefu, S. , Tran, D. D. , Yano, H. , & Kim, A. H. (2015). A CDC20‐APC/SOX2 signaling axis regulates human glioblastoma stem‐like cells. Cell Reports, 11(11), 1809–1821. 10.1016/j.celrep.2015.05.027 26074073 PMC4481182

[acel13251-bib-0027] Ministry of Health (2010a). Standard growth chart of a Japanese child (height, 2010). Labour and Welfare http://www.mhlw.go.jp/file/04‐Houdouhappyou‐11901000‐Koyoukintoujidoukateikyoku‐Soumuka/hyou2.xls

[acel13251-bib-0028] Ministry of Health (2010b). Standard growth chart of a Japanese child (weight, 2010). Labour and Welfare http://www.mhlw.go.jp/file/04‐Houdouhappyou‐11901000‐Koyoukintoujidoukateikyoku‐Soumuka/hyou1_1.xls

[acel13251-bib-0029] Miyamoto, T. , Porazinski, S. , Wang, H. , Borovina, A. , Ciruna, B. , Shimizu, A. , Kajii, T. , Kikuchi, A. , Furutani‐Seiki, M. , & Matsuura, S. (2011). Insufficiency of BUBR1, a mitotic spindle checkpoint regulator, causes impaired ciliogenesis in vertebrates. Human Molecular Genetics, 20(10), 2058–2070. 10.1093/hmg/ddr090 21389084

[acel13251-bib-0030] Nagai, T. , Ibata, K. , Park, E. S. , Kubota, M. , Mikoshiba, K. , & Miyawaki, A. (2002). A variant of yellow fluorescent protein with fast and efficient maturation for cell‐biological applications. Nature Biotechnology, 20(1), 87–90. 10.1038/nbt0102-87 11753368

[acel13251-bib-0031] Nishino, T. , Rago, F. , Hori, T. , Tomii, K. , Cheeseman, I. M. , & Fukagawa, T. (2013). CENP‐T provides a structural platform for outer kinetochore assembly. EMBO Journal, 32(3), 424–436. 10.1038/emboj.2012.348 23334297 PMC3567495

[acel13251-bib-0032] Niwa, H. , Yamamura, K. , & Miyazaki, J. (1991). Efficient selection for high‐expression transfectants with a novel eukaryotic vector. Gene, 108(2), 193–199. 1660837 10.1016/0378-1119(91)90434-d

[acel13251-bib-0033] Pfau, S. J. , Silberman, R. E. , Knouse, K. A. , & Amon, A. (2016). Aneuploidy impairs hematopoietic stem cell fitness and is selected against in regenerating tissues in vivo. Genes & Development, 30(12), 1395–1408. 10.1101/gad.278820.116 27313317 PMC4926863

[acel13251-bib-0034] Ran, F. A. , Hsu, P. D. , Wright, J. , Agarwala, V. , Scott, D. A. , & Zhang, F. (2013). Genome engineering using the CRISPR‐Cas9 system. Nature Protocols, 8(11), 2281–2308. 10.1038/nprot.2013.143 24157548 PMC3969860

[acel13251-bib-0035] Rehen, S. K. , Yung, Y. C. , McCreight, M. P. , Kaushal, D. , Yang, A. H. , Almeida, B. S. , & Chun, J. (2005). Constitutional aneuploidy in the normal human brain. Journal of Neuroscience, 25(9), 2176–2180. 10.1523/JNEUROSCI.4560-04.2005 15745943 PMC6726097

[acel13251-bib-0036] Santaguida, S. , & Amon, A. (2015). Short‐ and long‐term effects of chromosome mis‐segregation and aneuploidy. Nature Reviews Molecular Cell Biology, 16(8), 473–485. 10.1038/nrm4025 26204159

[acel13251-bib-0037] Snape, K. , Hanks, S. , Ruark, E. , Barros‐Núñez, P. , Elliott, A. , Murray, A. , Lane, A. H. , Shannon, N. , Callier, P. , Chitayat, D. , Clayton‐Smith, J. , FitzPatrick, D. R. , Gisselsson, D. , Jacquemont, S. , Asakura‐Hay, K. , Micale, M. A. , Tolmie, J. , Turnpenny, P. D. , Wright, M. , … Rahman, N. (2011). Mutations in CEP57 cause mosaic variegated aneuploidy syndrome. Nature Genetics, 43(6), 527–529. 10.1038/ng.822 21552266 PMC3508359

[acel13251-bib-0038] Tadaka, S. , Saigusa, D. , Motoike, I. N. , Inoue, J. , Aoki, Y. , Shirota, M. , Koshiba, S. , Yamamoto, M. , & Kinoshita, K. (2018). jMorp: Japanese multi omics reference panel. Nucleic Acids Research, 46(D1), D551–D557. 10.1093/nar/gkx978 29069501 PMC5753289

[acel13251-bib-0039] Takenouchi, T. , Yamaguchi, Y. , Tanikawa, A. , Kosaki, R. , Okano, H. , & Kosaki, K. (2015). Novel overgrowth syndrome phenotype due to recurrent de novo PDGFRB mutation. Journal of Pediatrics, 166(2), 483–486. 10.1016/j.jpeds.2014.10.015 25454926

[acel13251-bib-0040] Takenouchi, T. , Yoshihashi, H. , Sakaguchi, Y. , Uehara, T. , Honda, M. , Takahashi, T. , & Miyama, S. (2016). Hirschsprung disease as a yet undescribed phenotype in a patient with ARID1B mutation. American Journal of Medical Genetics Part A, 170(12), 3249–3252. 10.1002/ajmg.a.37861 27511161

[acel13251-bib-0041] Tian, W. , Li, B. , Warrington, R. , Tomchick, D. R. , Yu, H. , & Luo, X. (2012). Structural analysis of human Cdc20 supports multisite degron recognition by APC/C. Proceedings of the National Academy of Sciences of the United States of America, 109(45), 18419–18424. 10.1073/pnas.1213438109 23091007 PMC3494910

[acel13251-bib-0042] Vijg, J. , & Suh, Y. (2013). Genome instability and aging. Annual Review of Physiology, 75, 645–668. 10.1146/annurev-physiol-030212-183715 23398157

[acel13251-bib-0043] Wijshake, T. , Malureanu, L. A. , Baker, D. J. , Jeganathan, K. B. , van de Sluis, B. , & van Deursen, J. M. (2012). Reduced life‐ and healthspan in mice carrying a mono‐allelic BubR1 MVA mutation. PLoS Genetics, 8(12), e1003138 10.1371/journal.pgen.1003138 23300461 PMC3531486

[acel13251-bib-0044] Yost, S. , de Wolf, B. , Hanks, S. , Zachariou, A. , Marcozzi, C. , Clarke, M. , de Voer, R. M. , Etemad, B. , Uijttewaal, E. , Ramsay, E. , Wylie, H. , Elliott, A. , Picton, S. , Smith, A. , Smithson, S. , Seal, S. , Ruark, E. , Houge, G. , Pines, J. , … Rahman, N. (2017). Biallelic TRIP13 mutations predispose to Wilms tumor and chromosome missegregation. Nature Genetics, 49(7), 1148–1151. 10.1038/ng.3883 28553959 PMC5493194

[acel13251-bib-0045] Yu, H. (2007). Cdc20: a WD40 activator for a cell cycle degradation machine. Molecular Cell, 27(1), 3–16. 10.1016/j.molcel.2007.06.009 17612486

