# Supplementary material for: Premature aging syndrome showing random chromosome number instabilities with CDC20 mutation
Source: Aging Cell. 2020 Oct 23;19(11):e13251. doi: 10.1111/acel.13251 (PMC7681047; doi:10.1111/acel.13251)
Supplement: Supplementary file 1 [file ACEL-19-e13251-s001.pdf]

Supporting Information

Supplemental Text 1: Detailed patient clinical phenotypes

The patient is a 54-year-old XY female (II-1 in Figure 1a, body weight 33.4 kg [-2.4 SD], height 138.8 cm [-3.7 SD] at 48 years old). She was born healthy at 38-week gestation (birth weight 3,300 g [+1.4 SD], length 50.7 cm [+1.1 SD], head circumference 33.5 cm [+0.3 SD]), but several months after birth, the patient gradually showed growth retardation without mental retardation (Figure 1b). At 12 years old, she experienced surgical extrusion of streak gonads because of male pseudohermaphroditism. Up to 20 years old, she lost most of her hair and suffered from dry and atrophic skin with hyper- and hypo-pigmented macules (Figure 1c-e and Figure S1). At 26 years old, she developed progressive bilateral renal failure and started renal dialysis. At 28 years old, cataract extraction was performed due to bilateral cataracts. At 30 years old, she underwent kidney transplantation. At that time, both of the kidneys were retained but were highly atrophic and reduced in size. In her 30s, necrosis of the femoral head was indicated. At 48 years old, uterine endometrial carcinoma developed, which was resected with the ovary and greater omentum. Around 50 years old, she developed severe anemia because of bone marrow hypoplasia and started to receive monthly blood transfusions. At 53 years old, she was diagnosed with hypothyroidism. Additional findings include leg length discrepancy (the left leg is longer than the right), atrophy of the oral mucosa, nail deformities, low cardiac output, and kyphosis. The patient showed no neurodevelopmental, cognitive, memory, or other neurological defects. Her parents (I-1 and I-2 in Figure 1a) and younger sister (II-2 in Figure 1a) do not show such phenotypes.

Supplemental Text 2: Exome sequencing analyses and assessment of the identified genes

To identify the causative variants of the patient's disease, we performed exome sequencing using the peripheral blood genomic DNA of the patient and her parents. First, we checked rare variants in MVA syndrome-related genes and premature-aging-related genes in the patient genomic DNA with a minor allele frequency (MAF) < 0.03 in public databases including the Human Genetic Variation Database (Higasa et al., 2016), the Tohoku Medical Megabank Organization (ToMMo) 3.5KJPNv2 (Tadaka et al., 2018), Japanese Genotype-phenotype Archive (Kodama et al., 2015), 1000 Genomes Project (Genomes Project et al., 2015), and Exome Aggregation Consortium (Lek

et al., 2016). Several heterozygous rare variants in *RECQL4*, *FANCA*, *BRCA2,* and *FANCG*, known as the causative genes of Rothmund Thomson syndrome and Fanconi anemia, were identified (Table S5), but these diseases are autosomal-recessive diseases and their symptoms are far different from the ones of the patient. Thus, these variants are unlikely to be pathogenic. No rare variants with an MAF < 0.03 were identified in the following genes: *BUBR1(BUB1B)*, *CEP57*, *TRIP13*, *WRN*, *LMNA*, *BLM*, *ERCC8 (CSA)*, *XPA*, *ERCC3 (XPB)*, *XPC*, *ERCC2 (XPD)*, *DDB 2(XPE)*, *ERCC4 (XPF)*, *ERCC5(XPG)*, *ERCC6(CSB)*, *POLH (XPV)*, *ATM*, *GTF2H5 (TTDA)*, *ZMPSTE24*, *FANCB*, *FANCC*, *FANCD2*, *FANCE*, *FANCF*, *FANCI*, *BRIP1 (FANCJ)*, *FANCL*, *FANCM, PALB2 (FANCN)*, *RAD51C (FANCO)*, *SLX4 (FANCP)*, *RAD51 (FANCR)*, *BRCA1 (FANCS)*, *UBE2T (FANCT)*, *XRCC2 (FANCU)*, *MAD2L2 (FANCV)*, and *RFWD3 (FANCW)*.

Candidate variants were identified by the autosomal-dominant (AD) model (*de novo* mutations identified only in the patient) and the autosomal-recessive (AR) model (homozygous, hemizygous, or compound heterozygous rare variants identified in the patient). These variants were filtered with an MAF < 0.001 in the AD model, and an MAF < 0.03 in the AR model in public databases including the Human Genetic Variation Database (Higasa et al., 2016), the Tohoku Medical Megabank Organization integrative Japanese Genome Variation Database (Yamaguchi-Kabata et al., 2015), Japanese Genotype-phenotype Archive (Kodama et al., 2015) 1000 Genomes Project (Genomes Project et al., 2015), and Exome Aggregation Consortium (Lek et al., 2016). The filtered mutations and *in silico* predictions of protein damage using PolyPhen-2 (Adzhubei et al., 2010), SIFT (Kumar, Henikoff, & Ng, 2009). PROVEAN (Choi, Sims, Murphy, Miller, & Chan, 2012), Mutation Taster (Schwarz, Rodelsperger, Schuelke, & Seelow, 2010), and CADD scores (Rentzsch, Witten, Cooper, Shendure, & Kircher, 2019) are listed in Table S6. The AD model identified three heterozygous *de novo* germline mutations in *CDC20*, *ZBTB11*, and *TLCD2*. The AR model identified six homozygous (*KMT2E*, *DNAJB9, CENPT*, *ZFP90*, *MARVELD3* and *CNTNAP4*) and two compound heterozygous (*COL6A6* and *DNAH8*) pairs of rare variants and one hemizygous rare variant (*HCFC1*).

Among them, we considered the *CDC20* p.R286S mutation to be likely pathogenic for the following reasons: (1) CDC20 is a mitotic activator of APC/C ubiquitin ligase, which triggers the

metaphase–anaphase transition; (2) CDC20 function is negatively regulated by direct binding of BUBR1, a product of the known mosaic variegated aneuploidy (MVA)-causative gene *BUBR1*; (3) the R286 residue is well conserved among eukaryotes and localizes to the binding site of BUBR1 (Figure 2d-f).

ZBTB11 and *TLCD2*, the other variants identified by the AD model, are not likely to be causative genes for the following reasons: *ZBTB11*, which encodes the zinc-finger-containing transcription factor, has been reported to be a candidate causative gene of intellectual disability in human (Fattahi et al., 2018), and *Zbtb11* mutant zebrafish show abnormalities of craniofacial development and hydrocephalus and impaired neutrophil development and differentiation, but these phenotypes are not observed in the patient. In addition, *Zbtb11* expression is restricted in the developing embryo, myeloid lineage cells of hematopoietic systems, and the central nervous system (Keightley et al., 2017), indicating that this gene is not related to chromosome segregation; *TLCD2* encodes the Tram-Lag-CLN8 domain-containing membrane protein with six transmembrane helices. TLCD2 is expressed in various tissues, particularly in heart, muscle, liver, small intestine, and fat, and regulates membrane fluidity by reducing the turnover of polyunsaturated fatty acid-containing phospholipids (Ruiz et al., 2018), and therefore is not considered to be involved in chromosome segregation.

Among the candidate variants identified by the AR model, only the biallelic *CENPT* c.862+3G>C is considered to have a possible effect on chromosome segregation, because it encodes a member of the centromere-localizing proteins that is necessary for kinetochore assembly (Nishino et al., 2013). We found that the *CENPT* c.862+3G>C variant resulted in the in-frame deletion of exon 11 by affecting mRNA splicing (Figure S3). The c.862+3G>C variant did not disturb the subcellular localization of CENPT to the kinetochores (Figure S3). A recent report showed that biallelic *CENPT* mutations induced growth retardation, mental retardation, microcephaly, cone-rod dystrophy, and motor delay in humans, and aberrant splicing of *CENPT* with the in-frame deletion of a section of exon 12 did not induce MVA (Hung et al., 2017). Because our patient showed distinct clinical phenotypes, including MVA (Table S7), we concluded that the homozygous variants of *CENPT* found in the patient did not directly cause the

MVA and premature aging phenotypes. It remains undetermined whether clinical phenotypes are modified by the *CENPT* mutation in the patient.

Figure S1. Patient phenotypes

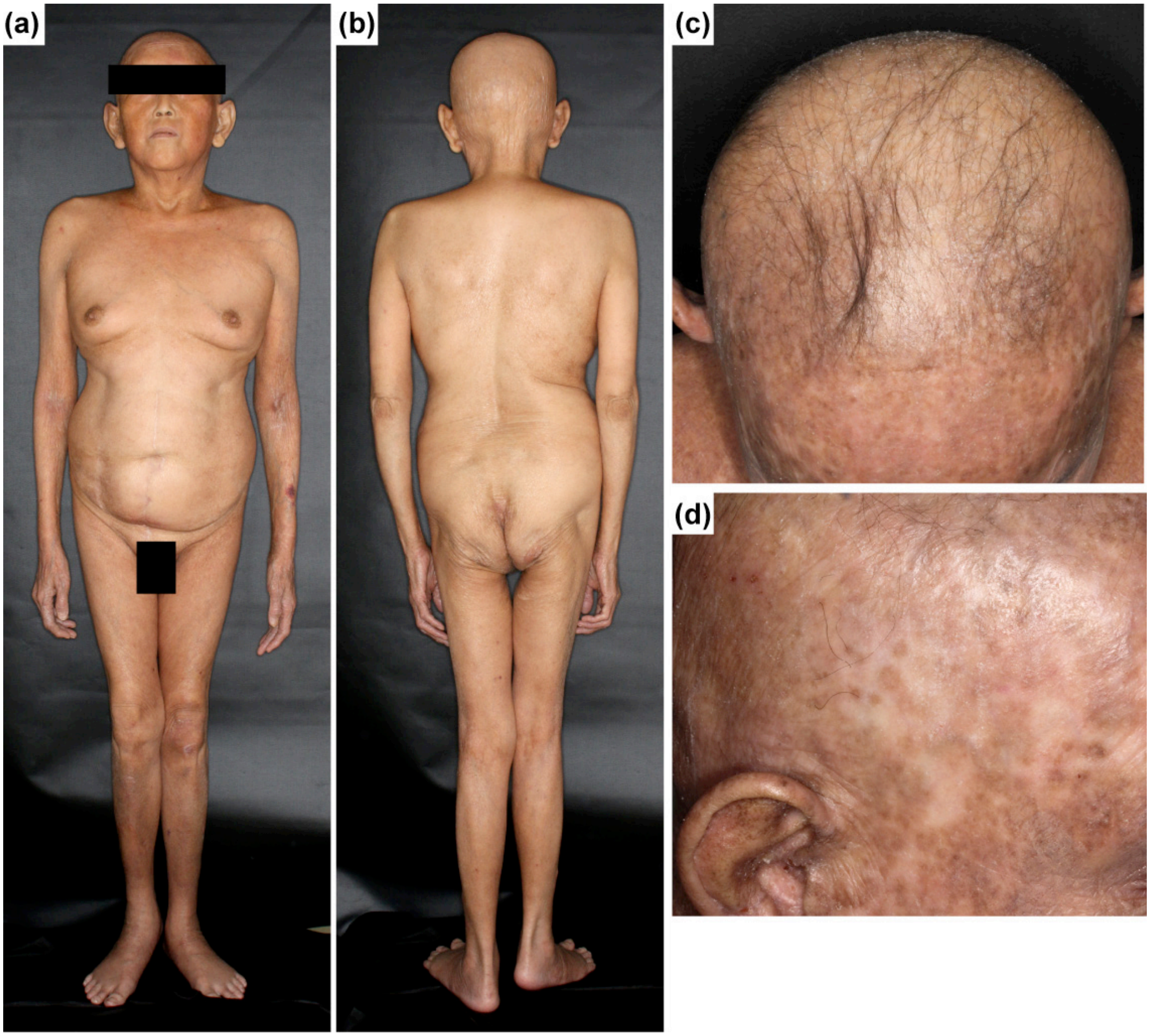

(a–d) Clinical photographs of the patient showing the whole body (a and b), loss of scalp hair (c), and multiple hyper- and hypo-pigmented macules (d).

Figure S2. Sanger sequencing chromatograms of the disease-causative candidates

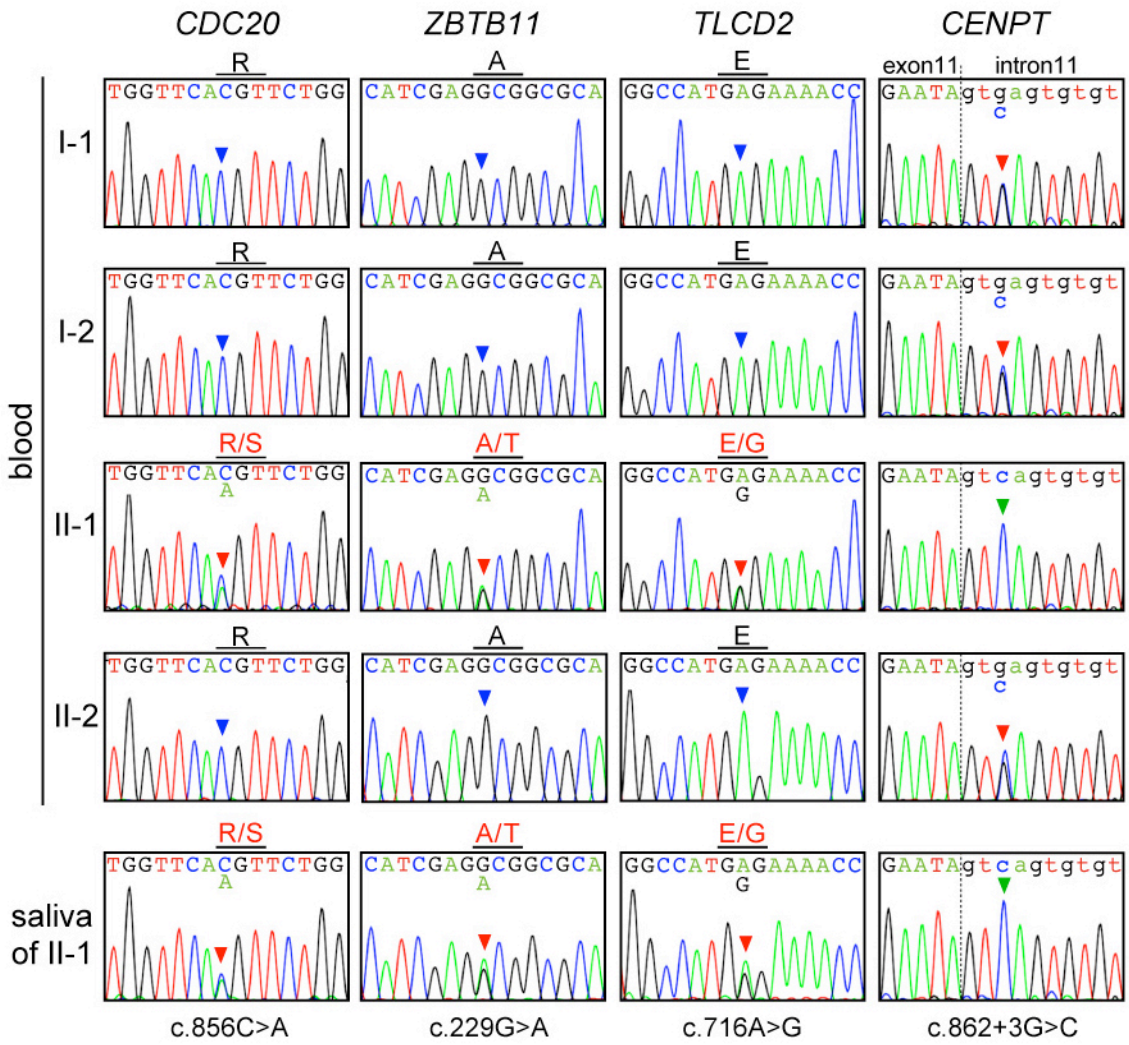

Sanger sequencing chromatograms of the genomic DNA from peripheral blood leukocytes from the patient (II-1), her parents (I-1 and I-2), her younger sister (II-2), and the patient saliva reveal disease-causative candidate variants in *CDC20* (c.856C>A, p.R286S), *ZBTB11* (c.229G>A, p.A77T), *TLCD2* (c.716A>G, pE239G), and *CENPT* (c.862+3G>C). Blue arrowheads, wild-type nucleotide; red arrowheads, heterozygous variant nucleotide; green arrowheads, homozygous variant nucleotide. The exon-intron boundary is indicated by dotted lines. Reference sequences are available from NCBI under the following accession numbers: *CDC20*, NM_001255.3/ NP_001246.2; *ZBTB11*, NM_014415.4/ NP_055230.2; *TLCD2*, NM_001164407.2/ NP_001157879.1; *CENPT*, NM_025082.4/ NP_079358.3

Figure S3. Characterization of the c.862+3G>C variant of *CENPT*

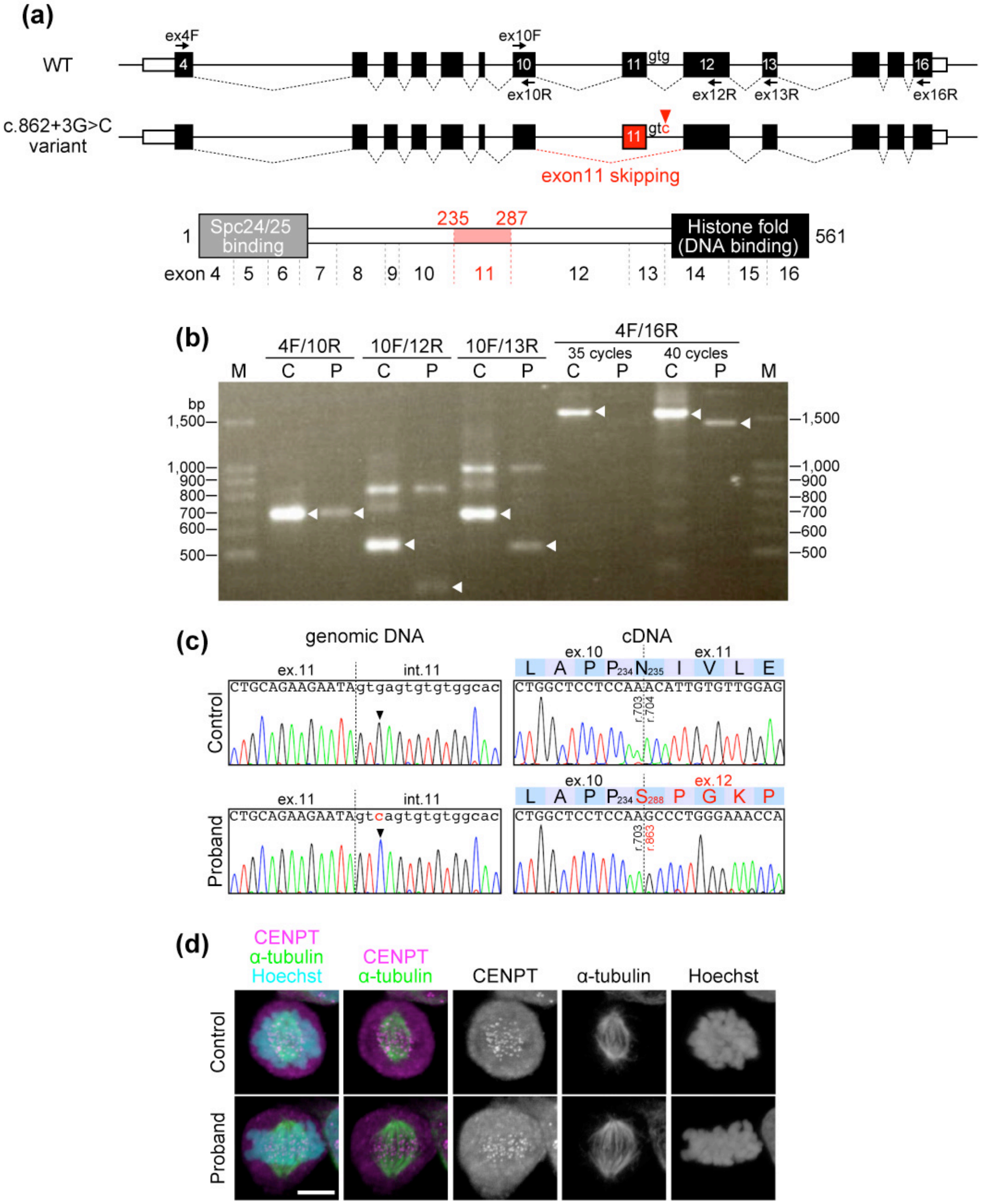

(a) Schematics of exon skipping in the c.862+3G>C variant of *CENPT*. Top, the exon/intron structure of the human WT and c.862+3G>C variant of *CENPT*. Coding and non-coding regions of exons are shown with filled and white boxes, respectively. The position of the c.862+3G>C variant is indicated by a red arrowhead. Positions of the primers used for the RT-PCR analyses are indicated with black arrows. Bottom, schematic of the functional domains

of CENPT. The gray box and the black box indicate the binding motif for Spc24/25 kinetochore proteins and DNA-binding histone-fold domain, respectively. The position of skipped exon 11 is indicated in red.
(b) PCR analyses of the WT and c.862+3G>C variant of *CENPT* from the cDNA. The cDNA was retrotranscribed from RNA extracted from the Epstein-Barr virus-transformed lymphoblastoid cell line (LCL) of a healthy control (C) and the patient (P). The PCR products amplified from *CENPT* cDNA using the primers shown in (a) are indicated by white arrowheads. The primer pairs of ex10F/12R, ex10F/13R, and ex4F/16R produced shortened PCR products from the patient's cDNA. M, molecular weight marker; bp, base pair.
(c) In-frame deletion of the entire exon11 in the c.862+3G>C variant of *CENPT*. Left, Sanger sequencing of *CENPT* in the LCL genomic DNA of the healthy control and the patient. The exon (ex.) -intron (int.) boundaries are shown with dotted lines. The positions of c.862+3 are indicated with arrowheads. Right, Sanger sequencing of *CENPT* in the cDNA of the LCLs of the healthy control and the patient. The boundary regions between the *CENPT* exon 10 and the following exons (exons 11 or 12) are shown with dotted lines. The majority of the *CENPT* cDNA of the patient's LCL was found to skip the whole of exon 11.
(d) Immunofluorescence images of CENPT (magenta), alpha-tubulin (green), and chromosomes (cyan, labeled with Hoechst 33342) in the mitotic phase of LCLs of the healthy control and the patient, showing kinetochore accumulation of CENPT. Images are representative of three independent experiments. Scale bar, 5 µm.

Figure S4. CRISPR/Cas9-mediated introduction of the *CDC20* p.R286S variant into HCT116 cells

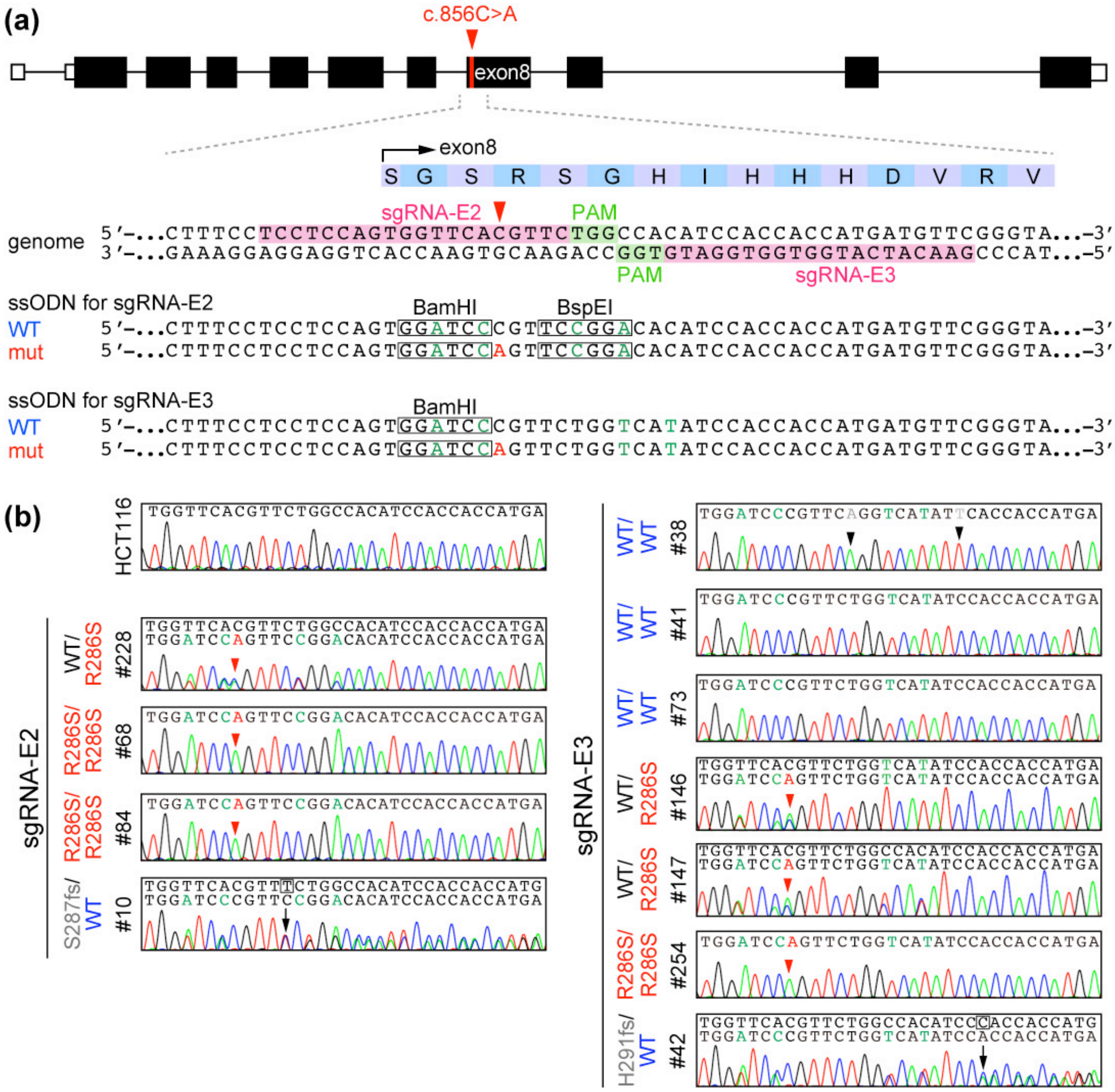

(a) Targeting strategy to introduce the *CDC20* c.856C>A (p.R286S) variant and wild-type silence variant into HCT116 cells. Schematic representation of the exon/intron structure of the human *CDC20* gene (filled box, coding exon; open box, non-coding exon; line, intron) represents the intra-genomic location of the c.856C>A variant in exon 8. The nucleotide sequences of the CRISPR/Cas9-targeted site at the intron 7-exon 8 boundary on the genomic DNA and single-strand oligonucleotides (ssODNs) for wild-type silence variant (WT), and the c.856C>A variant (mut) are shown. The binding sites of the single guide RNAs (sgRNA-E2 and -E3) and their protospacer adjacent motifs (PAM) are marked with pink and green on the genomic DNA sequence, respectively. The c.856C>A variant and the wild-type silent variants are indicated with red and green characters on the ssODN sequences, respectively. The endonuclease-recognition sites for the screening of variant clones are shown with open boxes.
(b) Sanger sequencing chromatograms of variant clones. The c.856C>A variant residues are indicated with red arrowheads. Silent variants are labeled with green characters in the nucleic acids. Single-base insertions causing frameshift (#10: c.859dupT [p.S287Ffs*8]; #42: c.871dupC [p.H291Pfs*4]) are labeled with black boxes and arrows.

Clone #38 contained two additional silent mutations (c.861T>A and c.870C>T labeled with gray characters and black arrowheads).

Figure S5. *In vitro* analyses of spindle assembly checkpoint failure and chromosome number instabilities in CRISPR-mediated *CDC20* p.R286S mutant cells

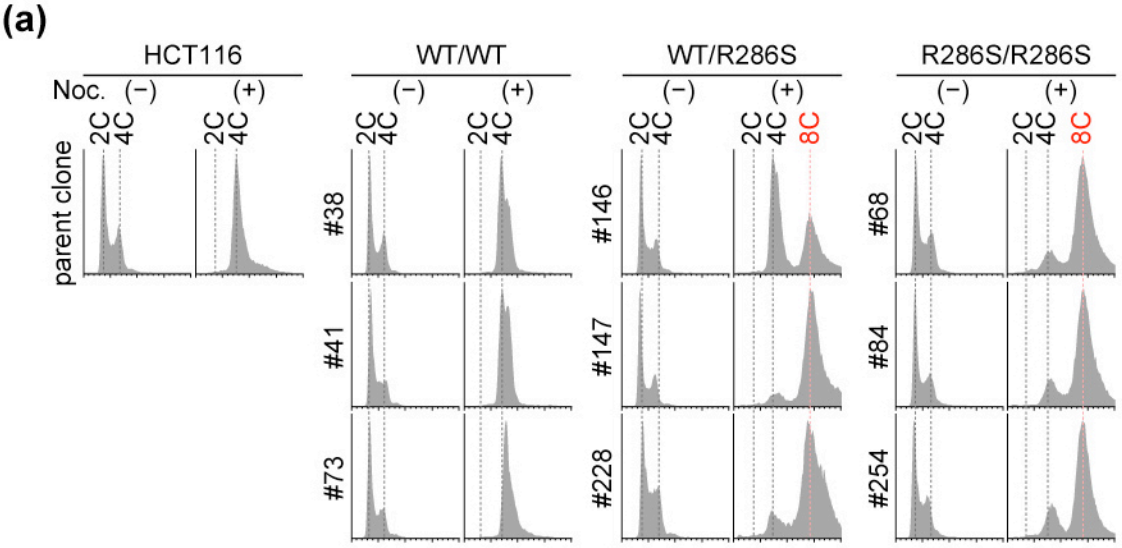

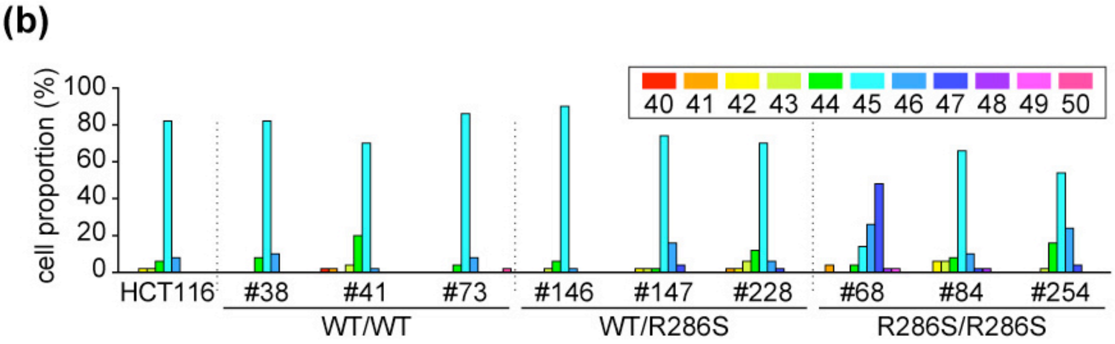

(a) Flow-cytometric analyses of the DNA contents in an HCT116 parent clone and its CRISPR-mediated mutant clones harboring the wild-type (WT) silence mutation or p.R286S mutation in CDC20 after 36 h of nocodazole (Noc.) treatment. X-axes indicate the nuclear DNA contents stained with 7-AAD on a linear scale. Representative histograms of three independent experiments are shown. 2C, diploid; 4C, tetraploid; 8C, octoploid. (b) Percentage of HCT116 cells showing normal and aberrant (shown in the indicated colors) number of chromosomes in the parent and mutant clones (50 metaphase spreads were counted for each clone).

Figure S6. Normal spindle assembly checkpoint in HCT116 cell clones bearing a monoallelic *CDC20* frameshift mutation

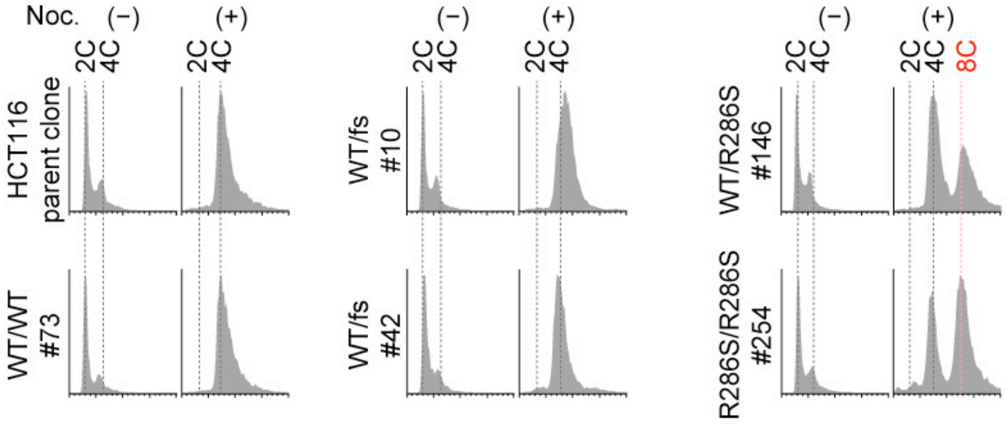

Flow-cytometric analyses of the DNA contents in CRISPR-mediated HCT116 mutant clones harboring a monoallelic frameshift variant of *CDC20* (WT/fs #10 and #42) compared to a parent clone, and CRISPR-mediated mutant clones harboring the wild-type silence mutation (WT/WT) and p.R286S mutation (WT/R286S and R286S/R286S harboring the mutation in monoallelic and biallelic manner, respectively) after 36 h of nocodazole (Noc.) treatment. X-axes indicate the nuclear DNA content stained with 7-amino-actinomycin D (7-AAD) on a linear scale. Histograms are representative of three independent experiments. 2C, diploid; 4C, tetraploid; 8C, octoploid.

Figure S7. Accumulation of the wild-type and p.R286S variant of CDC20 at metaphase kinetochores in HCT116 cells

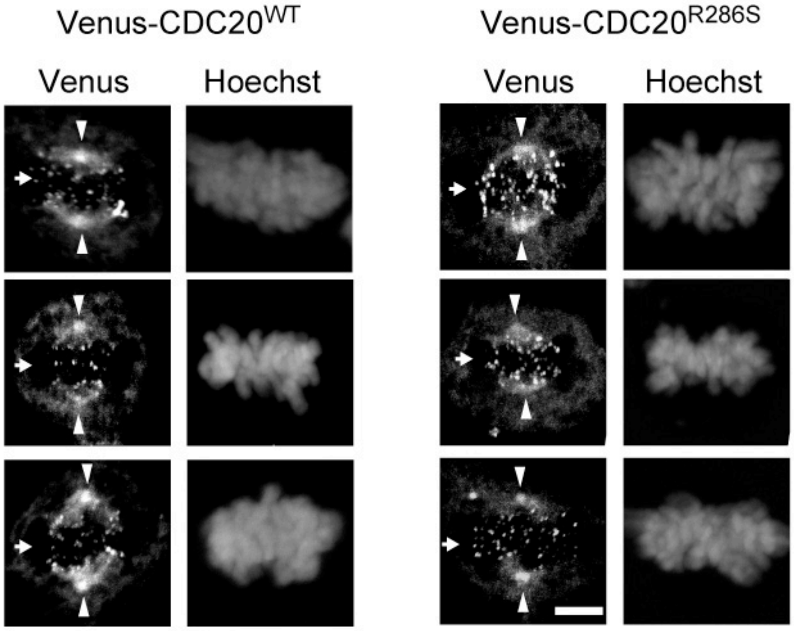

Confocal imaging of metaphase HCT116 cells expressing the Venus-fused wild-type (Venus-CDC20^{WT}) or p.R286S variant of CDC20 (Venus-CDC20^{R286S}). Accumulation of Venus signals to centrosomes and kinetochores are indicated by arrowheads and arrows. Images are representative of three independent experiments. Scale bar, 2 μm.

Figure S8. Immunoblotting of recombinant BUBR1 and CDC20 proteins used in the immunoprecipitation analyses

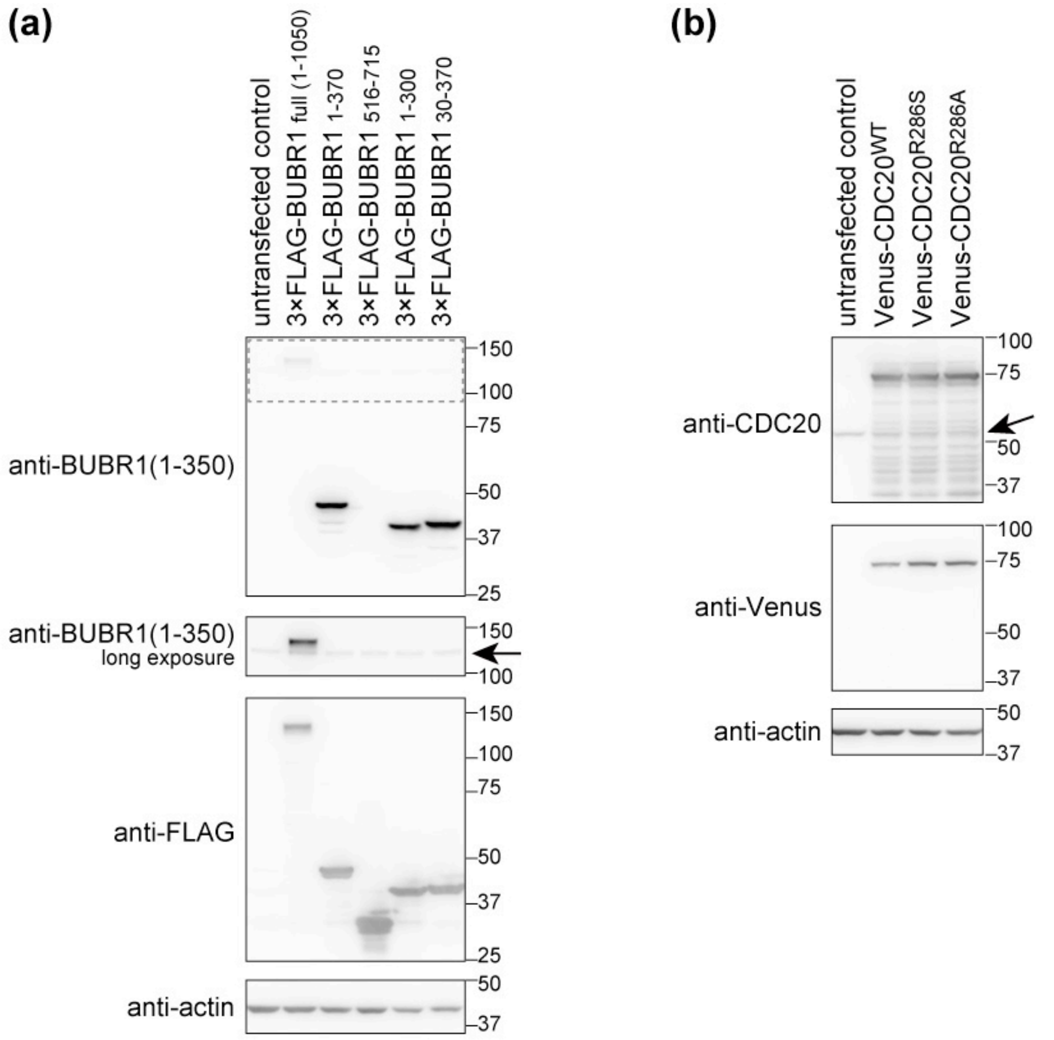

Immunoblotting of the extracts of 293T cells transfected with the expression plasmids for 3×FLAG-tagged BUBR1 proteins (a) or Venus-tagged CDC20 proteins (b). Transfected plasmids are shown on the top of the blots and antibodies used are shown on the left side of each blot. The dashed-square area of the upper panel in (a) was exposed for longer and the image is displayed in the second panel. Arrows indicate the endogenous proteins of BUBR1, and CDC20 in A and B, respectively. The 3×FLAG-BUBR1$_{516–715}$ fragment was detected by anti-FLAG antibody but not by anti-BUBR1 antibody because the anti-BUBR1 antibody recognizes only the N-terminal region of BUBR1 (aa1–350).

Figure S9. Uncropped images of the immunoblot shown in Figure 3e

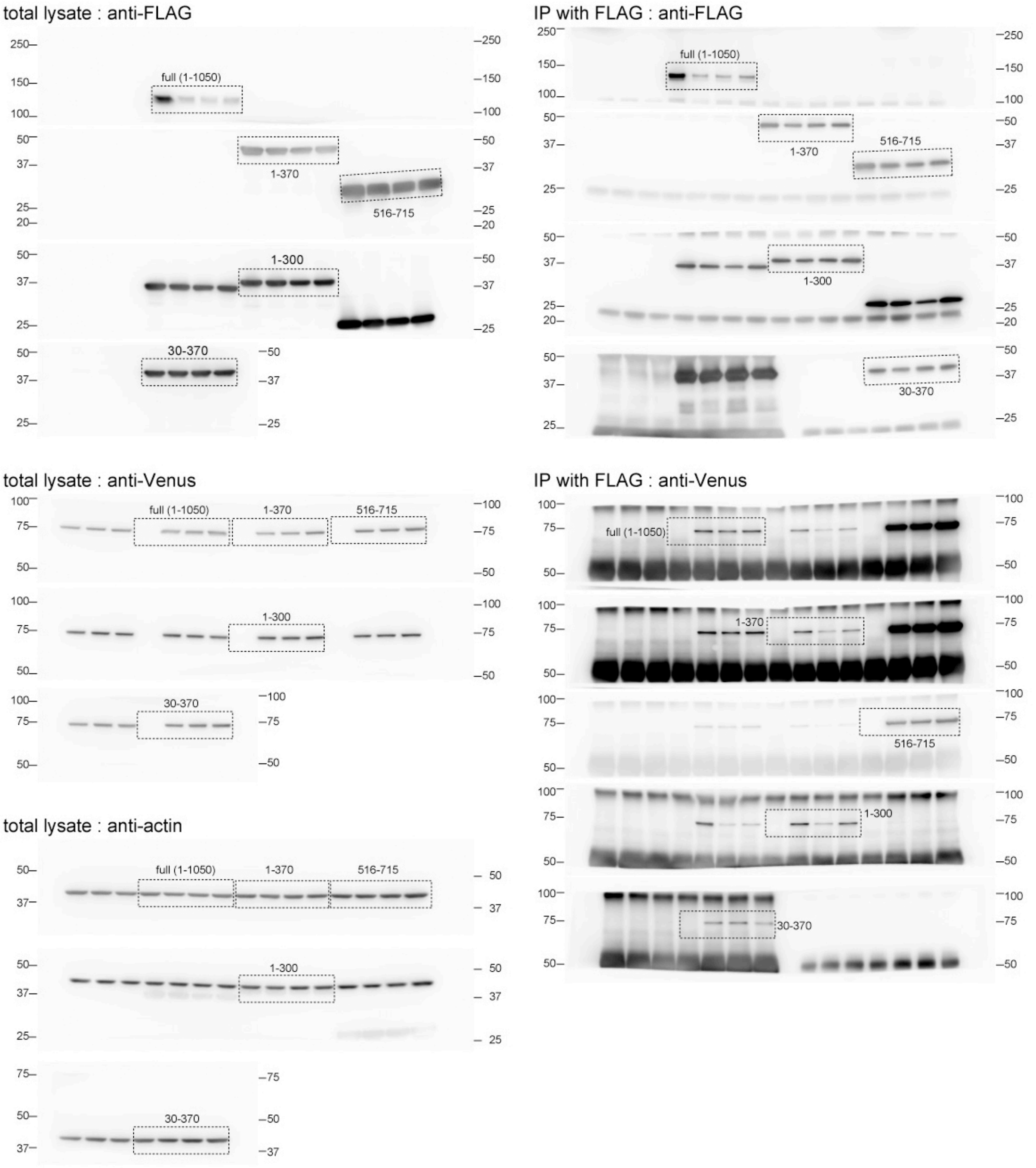

Table S1. Clinical features of the patient and other reported MVA syndromes

	MVA1	MVA2	MVA3	The patient
**Causative gene**	*BUBR1*	*CEP57*	*TRIP13*	*CDC20*
**MVA in peripheral blood**	9–78.3%	20–53%	+/–/ND	15%
**PCS in peripheral blood**	17–86.5%	0–14%	+/ND	6%
**Growth retardation (IUGR)**	15/17 (15/17)	7/7 (5/7)	4/6 (ND)	+ (–)
**Microcephaly**	14/17	2/7	3/6	–
**Seizures**	9/17	ND	2/6	–
**Mental retardation**	9/11	4/6	ND	–
**Skull anomalies**	ND	5/5	ND	–
**Rhizomelic shortening**	ND	5/7	ND	–
**Childhood cancer**	11/17[A]	0/7	6/6[B]	–
**Cataracts**	5/15	ND	ND	+
**Renal insufficiency**	2/17[C]	ND	1/6[D]	+
**Anemia**	1/17	ND	ND	+
**Hypothyroidism**	1/17	2/7	ND	+
**Hair loss**	ND	ND	ND	+
**Dry and atrophic skin**	ND	ND	ND	+
**Other skin abnormalities**	Hemangioma (1/17)	Single *café au lait* patch (1/7)<br>Acanthosis nigricans (1/7)	*Café au lait* patches (1/6)<br>Pigmentation on trunk (1/6)	Multiple hyper- and hypopigmentation<br>Nail deformity<br>Loss of subcutaneous fat
**Other remarkable phenotypes**	Dandy-Walker complex (7/17)<br>Multiple gastrointestinal neoplasia in adulthood (1/17)	Congenital heart disease (3/7)		XY female with complete female genitalia<br>Leg length discrepancy<br>Femur head necrosis<br>Kyphosis<br>Tonsil atrophy<br>Low cardiac output<br>Uterine endometrial carcinoma
**References**	Hanks et al. (2004)<br>Matsuura et al. (2006)<br>Rio Frio et al. (2010)<br>H. Ochiai et al. (2014)<br>Kato et al. (2017)<br>K. Ochiai et al. (2019)	Snape et al. (2011)<br>Pinson et al. (2014)<br>Brightman, Djaz, and Dauber (2018)<br>De la Torre-Garcia et al. (2019)	Yost et al. (2017)	this study

[A]Wilms tumor (6/11), ERMS (3/11), Wilms tumor and ERMS (2/11).
[B]Wilms tumor (6/6).
[C]multicystic kidney (1/2), impaired renal function (1/2)
[D]horseshoe kidney
Abbreviations: PCS, premature chromatid separation; MVA, mosaic variegated aneuploidy; IUGR, intrauterine growth retardation; ERMS, embryonal rhabdomyosarcoma; ND, not described.

Table S2. Clinical features of the patient and the major progeroid syndromes

	Hutchinson-Gilford progeria syndrome	Werner syndrome	The patient
**Causative gene**	*LMNA*	*WRN (RECQ3)*	*CDC20*
**Average life span (years)**	14.6	54	alive<br>(54-y-old, Feb 2020)
**Short stature**	+	+ (95%)*	+
**Thin limbs**	+	+ (98%)*	+
**Skin manifestations:**			
**dry skin**	+	–	+
**hyper- and hypopigmentation**	–	–	+
**scleroderma-like skin change**	+	+	–
**loss of subcutaneous fat**	+	+	+
**hair loss**	+	+	+
**nail dystrophy**	+	–	+
**skin ulcer**	–	+	–
**Bilateral cataracts**	–	+ (99%)*	+
**High voice tone**	+	+ (89%)*	–
**Arteriosclerosis**	+	+ (30%)*	–
**Neoplasm**	–	+ (44%)*	uterine endometrial carcinoma (48-y-old)
**Bilateral renal atrophy**	–	–	+
**Anemia**	–	–	+
**Other phenotypes**	stroke<br>lipodystrophy<br>progressive joint contracture<br>osteolysis<br>failure of secondary sexual development	type 2 diabetes mellitus (71%)*<br>soft tissue calcification (67%)*<br>osteoporosis (91%)*<br>hypogonadism (80%)*	XY female with complete female genitalia<br>leg length discrepancy<br>femur head necrosis<br>kyphosis<br>tonsil atrophy<br>low cardiac output<br>hypothyroidism
**Reference**	Gordon et al. (2019)	Oshima et al. (2016)	this study

*Percentages indicate incidence rates

Table S3. Karyotypes observed in the patient's peripheral blood mononuclear cells

Normal [212/250]		Aneuploidy [38/250]	
karyotype	n	karyotype	n
46, XY	212	43, XY, -6, -10, -20	1
		44, XY, -13, -20	1
		44, XY, -13, -22	1
		45, X, -Y	5
		45, X, -Y, -14, +21	1
		45, XY, -4	1
		45, XY, -4, -8, +13	1
		45, XY, -9	1
		45, XY, -10	1
		45, XY, -13	1
		45, XY, -19	1
		45, XY, -21	1
		47, XXY	1
		47, XY, +2	1
		47, XY, +6	2
		47, XY, +6, -13, +18	1
		47, XY, +7	1
		47, XY, +8	4
		47, XY, +13	3
		47, XY, +17	1
		47, XY, +18	2
		47, XY, +19	1
		47, XY, +21	1
		47, XY, +22	1
		48, XXY, +2	1
		48, XY, +7, +8	1
		48, XY, +8, +18	1

Table S4. Cytokinesis-block micronucleus assay of the patient and healthy controls

	age	sex	Micronuclei frequency (%)	mean ± SEM
Control 1	45	female	0.6	0.7 ± 0.2
Control 2	50	male	0.5	
Control 3	55	male	1.2	
Patient	53	XY female	2.5	-

Table S5. Rare variants in premature-aging-related genes in the patient

Gene symbol	RefSeq ID	Related disease	Inheritance	Zygosity of the variants	Mutation type	Nucleotide change	Amino acid change	Reference SNP ID	ToMMo 3.5KJPNv2 allele frequency	ToMMo 3.5KJPNv2 allele count/ number
RECQL4	NM_004260	Rothmund-Thomson syndrome	AR	Heterozygote	missense	c.1040G>A	p.R347H	rs757988816	0.0014	10/7,104
FANCG	NM_004629	Fanconi anemia	AR	Heterozygote	inframe insertion	c.373_375dupGTC	p.V125dup	rs750843326	0.0024	17/7,104
BRCA2	NM_000059	Fanconi anemia	AR	Heterozygote	missense	c.964A>C	p.K322Q	rs11571640	0.0135	96/7,104
FANCA	NM_000135	Fanconi anemia	AR	Heterozygote of two missense variants on one allele	missense	c.1049G>A	p.R350Q	rs199967286	0.0073	52/7,104
					missense	c.1844C>T	p.P615L	rs997083097	NA	NA

Abbreviations: AR, autosomal recessive; SNP, single nucleotide polymorphism; NA, not available.

Table S6. *De novo* mutations and rare homozygous or compound heterozygous variants identified by exome sequencing

	Gene symbol	RefSeq ID	Mutation type	Nucleotide change	Amino acid change	Reference SNP ID	PolyPhen-2 HumDiv [B] score (prediction)	SIFT [C] score (prediction)	PROVEAN [D] score (prediction)	Mutation Taster [E]	CADD score [F]	ToMMo 3.5kJPNv2 allele frequency	ToMMo 3.5kJPNv2 allele count/ number
AD model	***CDC20***	NM_001255	hetero, *de novo* missense	c.856C>A	p.R286S	NA	0.902 (possibly damaging)	0.037 (damaging)	-4.43 (deleterious)	disease-causing	24.7	NA	NA
	ZBTB11	NM_014415	hetero, *de novo* missense	c.229G>A	p.A77T	rs758014364	0.979 (probably damaging)	0.011 (damaging)	-0.66 (neutral)	disease-causing	24.2	NA	NA
	TLCD2	NM_001164407	hetero, *de novo* missense	c.716A>G	p.E239G	NA	0.081 (benign)	0.044 (damaging)	-1.37 (neutral)	polymorphism	15.85	NA	NA
AR model	***KMT2E***	NM_182931	homo, missense	c.1700T>C	p.I567T	rs573946129	0.993 (probably damaging)	0.007 (damaging)	-0.62 (neutral)	disease-causing	26.7	0.0004	3/7,104
	DNAJB9	NM_012328	homo, missense	c.440G>T	p.G147V	rs780779662	NA	0.498 (tolerated)	-0.25 (neutral)	polymorphism	12.65	0.0004	3/7,104
	CENPT	NM_025082	homo, splicing	c.862+3G>C	p.N235_N287del[A]	NA	NA	NA	NA	disease-causing	23.8	0.0004	3/7,104
	ZFP90	NM_133458	homo, missense	c.26C>T	p.A9V	rs139638624	0.000 (benign)	0.282 (tolerated)	-0.34 (neutral)	polymorphism	15.83	0.0101	72/7,104
	MARVELD3	NM_001017967	homo, missense	c.1114G>C	p.G372R	rs141358444	0.966 (probably damaging)	0.365 (tolerated)	-1.88 (neutral)	polymorphism	10.82	0.0008	6/7,104
	CNTNAP4	NM_138994	homo, missense	c.1850T>C	p.V617A	NA	0.002 (benign)	0.034 (damaging)	-1.04 (neutral)	polymorphism	15.13	NA	NA
	HCFC1	NM_005334	hemi, missense	c.4442C>T	p.T1481M	rs199798029	0.923 (possibly damaging)	0.015 (damaging)	-1.49 (neutral)	polymorphism	22.8	0.0081	45/5,554
	COL6A6	NM_001102608	compound hetero, missense	c.6191C>T	p.A2064V	rs1272434256	0.999 (probably damaging)	0.005 (damaging)	-3.17 (deleterious)	disease-causing	28	0.0018	13/7,104
				c.6502C>T	p.R2168C	rs193270756	0.279 (benign)	0.001 (damaging)	-4.76 (deleterious)	disease-causing	25.7	0.0041	29/7,104
	DNAH8	NM_001206927	compound hetero, missense	c.10418C>G	p.T3473S	rs182442615	0.000 (benign)	0.133 (tolerated)	-0.78 (neutral)	disease-causing	10.33	0.0014	10/7,104
				c.13069A>G	p.K4357E	rs139250593	0.002 (benign)	1.000 (tolerated)	-0.60 (neutral)	disease-causing	21.2	0.0021	15/7,104

Abbreviations: AD, autosomal dominant; AR, autosomal recessive; SNP, single nucleotide polymorphism; NA, not available.

[A]This amino acid change was confirmed as shown in Figure S3 in this paper

[B]http://genetics.bwh.harvard.edu/pph2/ (Adzhubei et al., 2010)

[C]http://provean.jcvi.org/genome_submit_2.php (Kumar et al., 2009)

[D]http://provean.jcvi.org/genome_submit_2.php (Choi et al., 2012)

[E]http://www.mutationtaster.org (Schwarz et al., 2010)

[F]https://cadd.gs.washington.edu (Rentzsch et al., 2019)

Table S7. Comparison of the phenotypes between the patient in this study and *CENPT* mutant patients

	***CENPT* mutant patients (Hung et al., 2017)**	**Study patient**
**Growth retardation**	+ (9/10)*	+
**Retinoblastoma**	+ (3/10)*	–
**Cone-rod dystrophy**	+ (6/10)*	–
**Nystagmus**	+ (5/10)*	–
**Microcephaly**	+ (10/10)*	–
**Intellectual disability**	+ (8/10)*	–
**Motor delay**	+ (7/10)*	–
**Spasticity**	+ (7/10)*	–
**Cataracts**	ND	+
**Kidney failure**	ND	+
**Anemia**	ND	+
**Hair loss**	ND	+
**Dry and atrophic skin**	ND	+

*Incidence rate. ND, not described.

Table S8. Primer sequences used in this study

PCR primers used for Sanger sequencing of genomic DNA

CDC20 ex8F	CAGTGGTTTTTGTTGGTCTATGG
CDC20 ex8R	GGTGGGGTCAGCAGGTACTA
TLCD2 ex4F	TCTTCAGGCCTGGGTTATTG
TLCD2 ex4R	GCATGGAAGACCCTCATCTC
ZBTB11 ex1F	CCTTGCCTTGCGTCACTT
ZBTB11 ex1R	CTCCGACTCGTGGGTACG
CENPT ex11+int11F	ATCTCCAGGTGACAGCCTCA
CENPT ex11+int11R	CATTGACCTCCTCTGCCTCT

PCR primers used for RT-PCR of *CENPT* gene and following Sanger sequencing

CENPT ex4F	CACAACCCTGACAGCGACT
CENPT ex10F	CACCTCTTCAGCCACAGTCA
CENPT ex10R	GGAGGAGCCAGGGAAGTATC
CENPT ex12R	CCCTCTGCTTCTGTCACCTC
CENPT ex13R	GGCTGGCTCAAGAAACTGAT
CENPT ex16R	CACGTGCAGTGAGACTTGGT

PCR primers used for mutagenesis of pCAGGS-Venus-hCDC20

CDC20-QC-T	CTATATCCTGTCCAGTGGTTCAAGTTCTGGCCACATCCAC
CDC20-QC-B	GTGGATGTGGCCAGAACTTGAACCACTGGACAGGATATAG
CDC20(KOD-R286A)-F	GCTTCTGGCCACATCCACCACCATGATG
CDC20(KOD-R286A)-R	TGAACCACTGGACAGGATATAGC

PCR primers used for cloning of truncated BUBR1 fragments

BamHI-M1-F	AAAGGATCCATGGCGGCGGTGAAGAAGGAA
BamHI-Q30-F	AAAGGATCCCAACCTTTAAGGCAAGGGCG
BamHI-Q516-F	AAAGGATCCCAGGAACAACCTCATTCTAAAG
NotI-M300-R	AAAGCGGCCGCTCACATGGGGGGTGCTATCCATGG
NotI-K370-R	AAAGCGGCCGCTCACTTTCTGGTGCTTAGGATGTG
NotI-Q715-R	AAAGCGGCCGCTCATTCTGAAGTCTCATTAGTAAGTTC

Supplemental References

Adzhubei, I. A., Schmidt, S., Peshkin, L., Ramensky, V. E., Gerasimova, A., Bork, P., . . . Sunyaev, S. R. (2010). A method and server for predicting damaging missense mutations. *Nat Methods, 7*(4), 248-249. doi:10.1038/nmeth0410-248

Brightman, D. S., Djaz, S., & Dauber, A. (2018). Mosaic variegated aneuploidy syndrome caused by a CEP57 mutation diagnosed by whole exome sequencing. *Clinical Case Reports, 6*(8), 1531-1534. doi:10.1002/ccr3.1655

Choi, Y., Sims, G. E., Murphy, S., Miller, J. R., & Chan, A. P. (2012). Predicting the functional effect of amino acid substitutions and indels. *PLoS One, 7*(10), e46688. doi:10.1371/journal.pone.0046688

De la Torre-Garcia, O., Mar-Aldama, R., Salgado-Sangri, R., Diaz-Gomez, N., Bonilla-Arcaute, L., Diaz-Ponce-Medrano, J., . . . Martinez-Hernandez, A. (2019). A homozygous CEP57 c.915_925dupCAATGTTCAGC mutation in a patient with mosaic variegated aneuploidy syndrome with rhizomelic shortening in the upper and lower limbs and a narrow thorax. *Eur J Med Genet, 62*(3), 195-197. doi:10.1016/j.ejmg.2018.07.013

Fattahi, Z., Sheikh, T. I., Musante, L., Rasheed, M., Taskiran, II, Harripaul, R., . . . Najmabadi, H. (2018). Biallelic missense variants in ZBTB11 can cause intellectual disability in humans. *Hum Mol Genet, 27*(18), 3177-3188. doi:10.1093/hmg/ddy220

Genomes Project, C., Auton, A., Brooks, L. D., Durbin, R. M., Garrison, E. P., Kang, H. M., . . . Abecasis, G. R. (2015). A global reference for human genetic variation. *Nature, 526*(7571), 68-74. doi:10.1038/nature15393

Gordon LB, Brown WT, Collins FS. (January 17, 2019) Hutchinson-Gilford Progeria Syndrome In: GeneReviews at GeneTests; Medical Genetics Information Resource (database online). Copyright, University of Washington, Seatle, 1997-2019; Available at https://www.ncbi.nlm.nih.gov/books/NBK1121/.

Hanks, S., Coleman, K., Reid, S., Plaja, A., Firth, H., Fitzpatrick, D., . . . Rahman, N. (2004). Constitutional aneuploidy and cancer predisposition caused by biallelic mutations in BUB1B. *Nat Genet, 36*(11), 1159-1161. doi:10.1038/ng1449

Higasa, K., Miyake, N., Yoshimura, J., Okamura, K., Niihori, T., Saitsu, H., . . . Matsuda, F. (2016). Human genetic variation database, a reference database of genetic variations in the Japanese population. *J Hum Genet, 61*(6), 547-553. doi:10.1038/jhg.2016.12

Hung, C. Y., Volkmar, B., Baker, J. D., Bauer, J. W., Gussoni, E., Hainzl, S., . . . Bodamer, O. A. (2017). A defect in the inner kinetochore protein CENPT causes a new syndrome of severe growth failure. *PLoS One, 12*(12), e0189324. doi:10.1371/journal.pone.0189324

Kato, M., Kato, T., Hosoba, E., Ohashi, M., Fujisaki, M., Ozaki, M., . . . Kurahashi, H. (2017). PCS/MVA syndrome caused by an Alu insertion in the BUB1B gene. *Hum Genome Var, 4*, 17021. doi:10.1038/hgv.2017.21

Keightley, M. C., Carradice, D. P., Layton, J. E., Pase, L., Bertrand, J. Y., Wittig, J. G., . . . Lieschke, G. J. (2017). The Pu.1 target gene Zbtb11 regulates neutrophil development through its integrase-like HHCC zinc finger. *Nat Commun, 8*, 14911. doi:10.1038/ncomms14911

Kodama, Y., Mashima, J., Kosuge, T., Katayama, T., Fujisawa, T., Kaminuma, E., . . . Nakamura, Y. (2015). The DDBJ Japanese Genotype-phenotype Archive for genetic and phenotypic human data. *Nucleic Acids Res, 43*(Database issue), D18-22. doi:10.1093/nar/gku1120

Kumar, P., Henikoff, S., & Ng, P. C. (2009). Predicting the effects of coding non-synonymous variants on protein function using the SIFT algorithm. *Nat Protoc, 4*(7), 1073-1081. doi:10.1038/nprot.2009.86

Lek, M., Karczewski, K. J., Minikel, E. V., Samocha, K. E., Banks, E., Fennell, T., . . . Exome Aggregation, C. (2016). Analysis of protein-coding genetic variation in 60,706 humans. *Nature, 536*(7616), 285-291. doi:10.1038/nature19057

Matsuura, S., Matsumoto, Y., Morishima, K., Izumi, H., Matsumoto, H., Ito, E., . . . Kajii, T. (2006). Monoallelic BUB1B mutations and defective mitotic-spindle checkpoint in seven families with premature chromatid separation (PCS) syndrome. *Am J Med Genet A, 140*(4), 358-367. doi:10.1002/ajmg.a.31069

Nishino, T., Rago, F., Hori, T., Tomii, K., Cheeseman, I. M., & Fukagawa, T. (2013). CENP-T provides a structural platform for outer kinetochore assembly. *EMBO J, 32*(3), 424-436. doi:10.1038/emboj.2012.348

Ochiai, H., Miyamoto, T., Kanai, A., Hosoba, K., Sakuma, T., Kudo, Y., . . . Matsuura, S. (2014). TALEN-mediated single-base-pair editing identification of an intergenic mutation upstream of BUB1B as causative of PCS (MVA) syndrome. *Proc Natl Acad Sci U S A, 111*(4), 1461-1466. doi:10.1073/pnas.1317008111

Ochiai, K., Yamada, A., Kimoto, Y., Imamura, H., Ikeda, T., Matsukubo, M., . . . Moritake, H. (2019). Long-term remission of bilateral Wilms tumors that developed from

premature separation of chromatids/mosaic variegated aneuploidy syndrome due to bilateral nephrectomy and peritoneal dialysis. *Pediatr Blood Cancer*, e27804. doi:10.1002/pbc.27804

Oshima J, Martin GM, Hisama FM. (September 29, 2016) Werner Syndrome. In: GeneReviews at GeneTests; Medical Genetics Information Resource (database online). Copyright, University of Washington, Seatle, 1997-2019; Available at https://www.ncbi.nlm.nih.gov/books/NBK1514/.

Pinson, L., Mannini, L., Willems, M., Cucco, F., Sirvent, N., Frebourg, T., . . . Musio, A. (2014). CEP57 mutation in a girl with mosaic variegated aneuploidy syndrome. *Am J Med Genet A, 164A*(1), 177-181. doi:10.1002/ajmg.a.36166

Rentzsch, P., Witten, D., Cooper, G. M., Shendure, J., & Kircher, M. (2019). CADD: predicting the deleteriousness of variants throughout the human genome. *Nucleic Acids Res, 47*(D1), D886-D894. doi:10.1093/nar/gky1016

Rio Frio, T., Lavoie, J., Hamel, N., Geyer, F. C., Kushner, Y. B., Novak, D. J., . . . Foulkes, W. D. (2010). Homozygous BUB1B mutation and susceptibility to gastrointestinal neoplasia. *N Engl J Med, 363*(27), 2628-2637. doi:10.1056/NEJMoa1006565

Ruiz, M., Bodhicharla, R., Svensk, E., Devkota, R., Busayavalasa, K., Palmgren, H., . . . Pilon, M. (2018). Membrane fluidity is regulated by the C. elegans transmembrane protein FLD-1 and its human homologs TLCD1/2. *Elife, 7*. doi:10.7554/eLife.40686

Schwarz, J. M., Rodelsperger, C., Schuelke, M., & Seelow, D. (2010). MutationTaster evaluates disease-causing potential of sequence alterations. *Nat Methods, 7*(8), 575-576. doi:10.1038/nmeth0810-575

Snape, K., Hanks, S., Ruark, E., Barros-Nunez, P., Elliott, A., Murray, A., . . . Rahman, N. (2011). Mutations in CEP57 cause mosaic variegated aneuploidy syndrome. *Nat Genet, 43*(6), 527-529. doi:10.1038/ng.822

Tadaka, S., Saigusa, D., Motoike, I. N., Inoue, J., Aoki, Y., Shirota, M., . . . Kinoshita, K. (2018). jMorp: Japanese Multi Omics Reference Panel. *Nucleic Acids Res, 46*(D1), D551-D557. doi:10.1093/nar/gkx978

Yamaguchi-Kabata, Y., Nariai, N., Kawai, Y., Sato, Y., Kojima, K., Tateno, M., . . . Nagasaki, M. (2015). iJGVD: an integrative Japanese genome variation database based on whole-genome sequencing. *Hum Genome Var, 2*, 15050. doi:10.1038/hgv.2015.50

Yost, S., de Wolf, B., Hanks, S., Zachariou, A., Marcozzi, C., Clarke, M., . . . Rahman, N. (2017). Biallelic TRIP13 mutations predispose to Wilms tumor and chromosome missegregation. *Nat Genet, 49*(7), 1148-1151. doi:10.1038/ng.3883
